# Cerebrospinal fluid outflow: a review of the historical and contemporary evidence for arachnoid villi, perineural routes, and dural lymphatics

**DOI:** 10.1007/s00018-020-03706-5

**Published:** 2021-01-11

**Authors:** Steven T. Proulx

**Affiliations:** Theodor Kocher Institute, University of Bern, Bern, Switzerland

**Keywords:** CSF, Clearance, Meningeal, Lymphatic vessels, Cranial nerves, Cribriform plate

## Abstract

Cerebrospinal fluid (CSF) is produced by the choroid plexuses within the ventricles of the brain and circulates through the subarachnoid space of the skull and spinal column to provide buoyancy to and maintain fluid homeostasis of the brain and spinal cord. The question of how CSF drains from the subarachnoid space has long puzzled scientists and clinicians. For many decades, it was believed that arachnoid villi or granulations, outcroppings of arachnoid tissue that project into the dural venous sinuses, served as the major outflow route. However, this concept has been increasingly challenged in recent years, as physiological and imaging evidence from several species has accumulated showing that tracers injected into the CSF can instead be found within lymphatic vessels draining from the cranium and spine. With the recent high-profile rediscovery of meningeal lymphatic vessels located in the dura mater, another debate has emerged regarding the exact anatomical pathway(s) for CSF to reach the lymphatic system, with one side favoring direct efflux to the dural lymphatic vessels within the skull and spinal column and another side advocating for pathways along exiting cranial and spinal nerves. In this review, a summary of the historical and contemporary evidence for the different outflow pathways will be presented, allowing the reader to gain further perspective on the recent advances in the field. An improved understanding of this fundamental physiological process may lead to novel therapeutic approaches for a wide range of neurological conditions, including hydrocephalus, neurodegeneration and multiple sclerosis.

## Introduction

Being arguably the most sophisticated organ, the brain requires ample protection from mechanical and molecular harm. The skull provides defense against physical insults, while the blood–brain barrier prevents foreign and potentially dangerous molecules from entering the brain. The cerebrospinal fluid (CSF) contained within the ventricles and subarachnoid space (SAS) provides another layer of support by creating a chemically and mechanically stable environment that is tightly controlled by additional blood–CSF barriers at the choroid plexus and arachnoid mater. Homeostasis in the production, circulation, and absorption of the CSF is therefore critical for normal brain function. Several diseases may arise from disturbances in the CSF system of which the most apparent are the ‘mechanical’ CSF disorders, e.g., the different forms of hydrocephalus and idiopathic intracranial hypertension. The presumed function of the CSF as a ‘waste-sink’ for toxic metabolites of the brain and of the SAS as a site of immune surveillance further suggests great importance of CSF homeostasis in relation to the development of neurodegenerative and neuroinflammatory diseases [1].

Many fundamental questions related to the production, circulation, and absorption of CSF remain unanswered. The studies considered to be the foundation of this field of research were first conducted around 150 years ago and since then, many different investigators have debated these topics and a satisfactory consensus has been difficult to reach [2–5]. Major reasons for this are that the CSF pathways, being located within the cranial cavity and spinal column, have been particularly difficult to access experimentally and most studies have therefore been based on ex vivo preparations or injections of exogenous tracers. Both techniques inherently introduce artifacts that may complicate interpretation of the data. It is also apparent that some of the concepts that have persisted to modern times were proposed by researchers who were not yet aware or failed to accurately consider the barriers of the central nervous system (CNS).

A widely cited value for CSF volume in humans is 140 mL and it is suggested that at least 500 mL of fluid is produced and cleared from the CNS every day [5]. However, it is most likely that this value for CSF volume is an underestimate. Recent MRI studies, while exhibiting significant variability between subjects, have provided average craniospinal CSF volume estimates ranging from 254 to 331 mL [6–8]. Regarding production, it is understood that this process is predominantly undertaken by the choroid plexus. However, additional roles for the blood–brain barrier in the parenchyma of the brain or ependymal cells lining the ventricles have been suggested [9, 10]. Circulation of CSF is complex but it is generally accepted that there is an overall directional flow with certain defined anatomical routes from the ventricles to the SAS to the sites of efflux. In addition, to-and-fro motions driven by arterial pulsations or respiratory movements are present [11–13]. On the other hand, a widely cited but controversial hypothesis, that of the existence of a “glymphatic” system, has proposed that a significant portion of CSF will enter the brain along perivascular spaces and flush through the brain parenchyma [14]. These questions regarding production and circulation, despite their intriguing nature, are outside the scope of this review and the reader is instead referred to several other recent reviews of these topics [15–18].

One very active area of debate relates to the clearance pathways of cerebrospinal fluid. In most organs of the body, lymphatic vessels are present within interstitial tissue and are responsible for draining fluid and proteins that have extravasated from the blood circulation [19, 20]. Even serous cavities, such as the peritoneal or pleural spaces, have lymphatic drainage through stomata that line the walls of these fluid spaces. The CNS on the other hand appears to be devoid of lymphatics not only within the brain or spinal cord parenchyma but also within the pia and arachnoid meningeal layers that directly align the CSF-filled SAS. Thus, alternative mechanisms for outflow have long been sought. The textbook understanding is that CSF will exit the SAS through specialized structures, called arachnoid villi or granulations, that extend into the venous sinuses in the dura mater [3, 5]. However, as will be evident in this review, no anatomical mechanism for how this process occurs has been settled upon and, surprisingly, no solid evidence from in vivo physiological studies has ever been produced that indicate that inert tracers will drain directly from the SAS to the blood. On the other hand, over a hundred studies in several different mammalian species have shown a role for the lymphatic vessels in draining tracers injected into CSF. Despite this direct evidence, the lymphatic contribution toward CSF outflow has consistently been minimalized. In fact, for a large portion of the twentieth century, lymphatic outflow of CSF was barely mentioned at all in textbooks on the subject. It was only after renewed efforts by investigators in the 1980s and 90s that a concept of dual outflow pathways, both venous and lymphatic, found wider acceptance [21–24].

The specific pathways through which the CSF can reach the lymphatic system are currently an area of great controversy. The bulk of the literature supports pathways through the cribriform plate and along cranial and spinal nerves to reach lymphatic vessels outside the CNS. However, in the last few years researchers have rediscovered a network of lymphatic vessels in the outermost meningeal layer, the dura mater, and have suggested that this could represent a more direct outflow route for CSF. This exciting finding has raised immediate questions regarding the potential mechanisms for this process, including how a barrier layer at the arachnoid mater that exists between the SAS and the dura mater could be traversed.

This review will first closely examine the historical and contemporary evidence for the three major proposed anatomical pathways for clearance of CSF from the cranium: arachnoid projections (villi or granulations), routes through foramina in the skull along cranial nerves (including the cribriform plate) and lymphatic vessels of the dura mater. Next, a description of the studies on CSF outflow from the spine will be presented. Subsequently, a detailed account of the physiological in vivo studies utilizing quantification of tracers and/or in vivo imaging will be provided. Finally, a summary of the limited evidence from humans and a discussion of the major open questions will attempt to set the stage for future work on this topic.

## Arachnoid villi and granulations

**Fig. 1:**
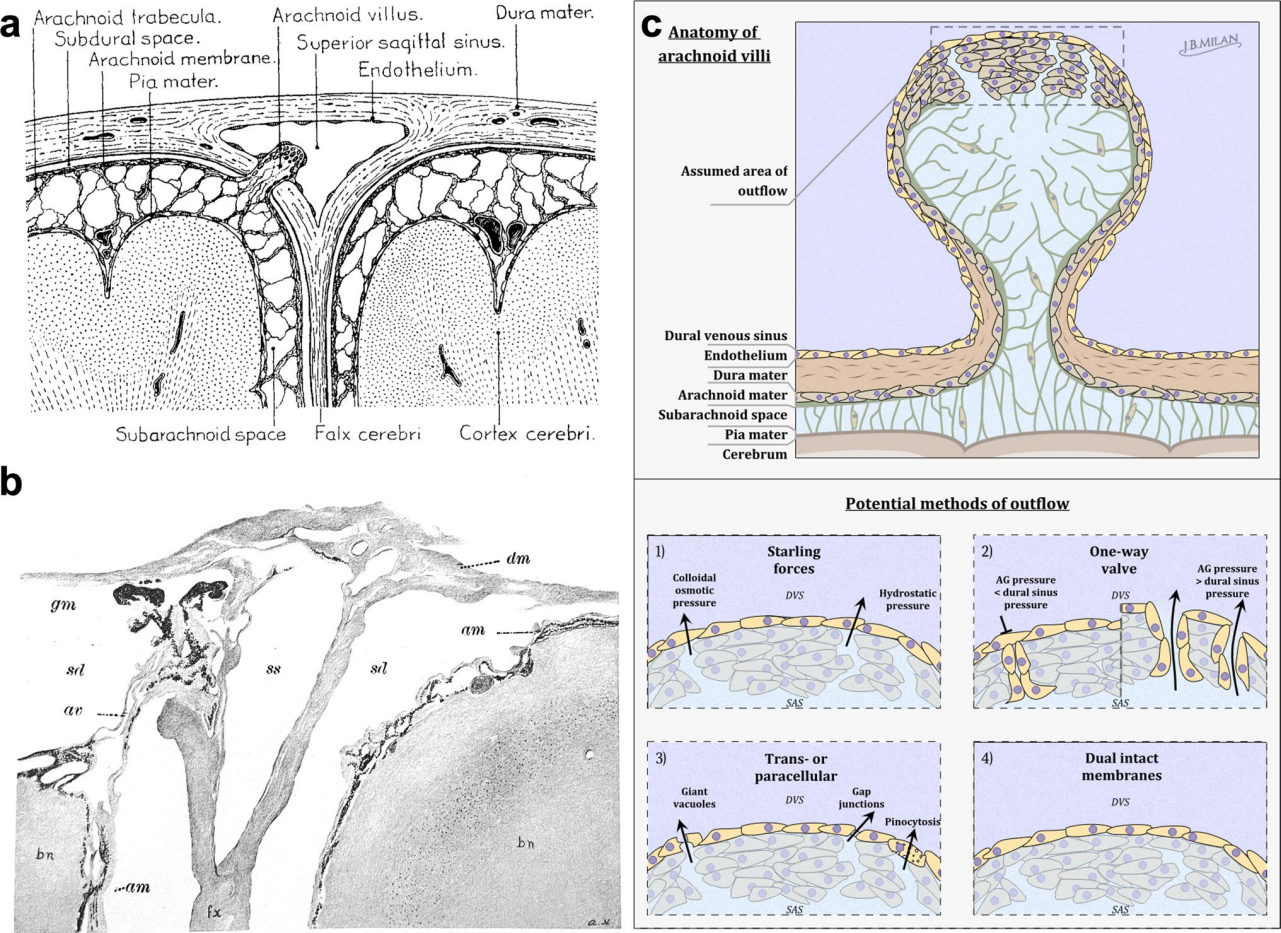
Arachnoid projections and the different models that have been proposed for CSF outflow to the dural venous sinus. **a** Lewis Weed’s rendering of an arachnoid villus (from [263]). The interior of the villus is composed of fluid spaces continuous with the subarachnoid space and contains fibers that that are connected with the arachnoid trabeculae. The villus projects through dural tissue to extend into the lumen of the superior sagittal sinus. A cap of arachnoid cells covered by an intact layer of endothelial cells is located at the terminus of the villus. **b** A reproduction of an image from Lewis Weed’s published report from 1914 [3] showing Prussian blue precipitate (gm, black granular material) within an arachnoid villus (av) extending toward the superior sagittal sinus (ss). Granules are seen in “isolated arachnoidal clumps near the sinus lumen”. The image is acquired from a cat that had been injected with 2% potassium ferrocyanide and iron ammonium citrate into the lumbar space under a pressure of 180 mm H_2_O for four hours. Note the large subdural space (sd) between the arachnoid mater (am) and dura mater (dm) which is an artifact of the tissue preparation. Weed also highlights the “absence of precipitate within the true dura tissue surrounding the arachnoidal elements” representing early evidence for the arachnoid barrier layer. **c** Various models attempting to provide a mechanism for outflow of CSF and solutes through arachnoid projections (villi or granulations) have been proposed. Panel 1 is Weed’s original conception based upon Starlings forces in which favorable hydrostatic and osmotic gradients would exist through the endothelial cell lining to facilitate CSF outflow to the dural venous sinus (DVS). Hugh Davson later challenged this concept as no osmotic forces would develop to allow macromolecular solutes, such as albumin, to leave the CSF [52]. Panel 2 is the proposal of arachnoid granulations (AG) as one-way valves, as conceived by Welch and Friedman [60]. When hydrostatic pressure gradients are favorable, endothelial-lined channels at the end of the projection are open for flow of fluid and macromolecules to the venous sinus. When the pressure in the sinus is above that of the SAS, then the channels are collapsed and no flow in either direction can occur. These endothelial-lined channels were later identified using electron microscopy as infoldings of the tissue that were not continuous with the SAS. Panel 3 are the various models that were proposed to explain how flow might occur through an intact layer of endothelial cells on the cap of the projections. Panel 4 represents the concept of a dual layer of intact cells on the cap of the projections, one layer comprised of arachnoid cells and the other of venous endothelium. In this scenario, no outflow of CSF would normally occur through these structures under homeostatic conditions. However, these layers may be distended and eventually disrupted under conditions of high intracranial pressure, allowing for a “safety valve” mechanism for rapid egress of CSF. Scheme by Joachim Birch Milan

The textbook model of CSF circulation states that arachnoid projections within the skull are the main CSF outflow site. Arachnoid projections are outcroppings of arachnoid tissue that extend through the dura into the major venous sinuses and the associated lacunae laterales and were first described in 1705 by the Italian anatomist Antonio Pacchioni [25]. There are considered to be two types of arachnoid projections: arachnoid villi and arachnoid granulations (aka Pacchionian bodies). The only major criteria to differentiate between the two types of tissue is their size, with villi described as microscopic and granulations grossly visible to the naked eye [26, 27]. For this reason, granulations are usually only described in larger animals and not in rodents in which many of the recent experiments have been performed. At the summit of the arachnoid projections, there is a layer of cells that comes into direct continuity with the venous sinuses of the dura [28, 29]. Thus, it would appear that these structures are ideally situated for drainage of CSF from the SAS to the venous blood (Fig. 1a).

A potential function for these structures remained elusive until the classic experiments of Key and Retzius published in 1875 [2]. In human cadavers and in living dogs and rabbits, injections of Richardson's blue dye were made and pathways to the veins were found through the projections of arachnoid tissue. As translated from the original German by Turner, “one can see that after injection into the subarachnoid space, the fluid fills the villi [...] then the fluid appears on their surface and from there it flows freely into the blood stream. The route is blocked only by two thin membranes. They are the surface of the villus and its dural sheath” [26]. Key and Retzius proposed that microscopic stomata were present between the endothelial cells of the dural sinus to allow the escape of the dye-filled fluid. Although criticized by later investigators for using high pressures for their injections which appeared to have led to rupture of the intervening membranes [30, 31], the concept by Key and Retzius of a drainage of CSF to veins through these specialized structures was highly influential.

One early point of controversy was that arachnoid granulations could not be found in lower vertebrates or in human infants [32]. Therefore, alternative mechanisms were sought. Harvey Cushing, working with Theodor Kocher in Switzerland, observed that mercury that had leaked from a rubber pressure bag inserted into the “subdural space” quickly reached the draining veins. He hypothesized that one-way valves must exist between the SAS and the dural sinuses [32]. Several other investigators around this time also concluded a direct transport of CSF injected dyes into the venous system by rapid detection within the draining veins of the neck or the urine [33–36]. For example, Dandy and Blackfan, detected a rapid absorption of phenolsulfonphthalein dye (phenol red) from the cerebral ventricles and the subarachnoid spaces and concluded that this transport to veins was a “diffuse process from the entire subarachnoid space” [37]. Meanwhile, others did not observe immediate outflow of dyes to the systemic circulation and were more inclined to believe that pathways leading to lymphatic vessels were the efflux mechanism [38, 39]. The discrepancies between these early observations likely reflect the differences in the properties of the tracer substances (lipid solubility, toxicity etc.) that were utilized, with some possessing the ability to cross CNS barriers and others not. Tracer properties will be discussed in more detail below in the section “In vivo quantitative studies”.

With the landmark work of Lewis Weed, published in 1914, the concept of drainage of CSF at the arachnoid villi that has largely persisted to the present day was established [3, 4, 31]. As this study has had the most influence on the textbook concept of the process of CSF outflow, it is worth examining this work in detail. In cats and dogs anesthetized with ether, a laminectomy was performed by a transverse cut of the lower thoracic or upper lumbar spinal cord and a cannula was inserted into the intrathecal space. Infusions at slightly above normal intracranial pressure (ICP) (130–180 mm H_2_O) of isotonic solutions of equal parts iron ammonium citrate and potassium ferrocyanide were then introduced into the spinal SAS at a slow rate. After various times of this infusion, the animals were killed. Weed then perfused the animals, via intracarotid injection, with a solution of formalin containing 1% hydrochloric acid. In this manner, the formalin fixed the brain and meninges, and the acid precipitated to form granules of Prussian blue from the previously introduced salts. Through this method, he avoided introducing particulate matter into the SAS which could have obstructed potential outflow pathways. In some animals, instead of perfusion, he severed the head intact and incubated the tissue for days in a solution of formaldehyde and hydrochloric acid. Weed found that after short periods of infusion, the Prussian blue granules were mainly located in the basal cisterns and the cavernous sinus, and, after longer periods of several hours, they were found concentrated above the cerebral hemispheres. At this location, he described microscopic structures of fine, web-like interlacing cords, which he termed arachnoid villi, that invaginated the arachnoid membrane into the dural superior sagittal sinus (Fig. 1b). As he was unable to locate any valve-like structures or stomata at this location through serial sections of the major dural sinuses, Weed argued that a process of filtration through an intact membrane at the cap of the arachnoid villi must be the transport mechanism. Weed also infused India ink in some animals but was unable to visualize its transport to peripheral tissues, instead the particulate material became enmeshed within arachnoid tissue in the SAS, as was also seen by other contemporary researchers [37, 40, 41].

Weed’s concept of outflow through arachnoid villi was not fully accepted by other researchers at that time. Working in dogs, Dandy found very few attachments of the cerebral hemispheres to the longitudinal, transverse, and circular sinuses [42]. When he physically separated the hemispheres from the dura and examined the animals four to six months later, he found no enlargement of SAS or ventricles, nor was any edema evident in the brain parenchyma. Weed also acknowledged in his original report that some earlier workers had reported toxic CNS effects of ferrocyanides [34, 43], although in his observations the prepared solutions did not exhibit any obvious toxicity. However, Howarth and Cooper, repeating Weed’s protocols much later in dogs, found severe toxic effects including muscular contractions, convulsions and even death [44]. Another early criticism of Weed’s methods was the fact that the tissue was only analyzed ex vivo after formalin fixation. By the time of examination, post mortem diffusion of the small molecular weight substrates had likely occurred, as suggested by several investigators [45–47]. During attempts to reproduce Weed’s work, it was found that Prussian blue had “impregnated the cranial bones, bones and ligaments of the spinal column and in cellular tissue surrounding blood vessels and nerves in the neck” [45]. Weed acknowledged this limitation himself in a later review [48] and investigators working in his laboratory attempted to address this issue by quickly freezing the superior sagittal sinus and associated tissue in situ through the use of a cold metal plate with subsequent freeze drying of the dissected sample [49]. They confirmed the presence of Prussian blue granules in the arachnoid villi and dural sinuses, but also reported that the granules were within the endothelial cells of veins crossing through the SAS. Thus, they suggested that this could represent a secondary means of CSF absorption. However, this viewpoint did not gain wide acceptance and further underscores the limitation of post mortem assessments of this process.

Weed and Hughson found that pressures within the superior sagittal sinus were usually lower than the pressure within the SAS [50]. However, it was also speculated that hydrostatic pressure gradients between the CSF and cranial venous sinuses are likely not always favorable to drive flow of CSF to the blood [48]. Therefore, Weed reasoned that osmotic forces according to the law of Starling must play a role in order to allow absorption to continue to the venous sinuses. In an experimental study, Weed showed that solutions containing high concentrations of serum proteins, gelatin or casein and, thus, with higher osmotic pressure, were absorbed more slowly than an isotonic crystalloid solution [51]. He concluded that effective force driving the normal process of absorption of the cerebrospinal fluid is a component of the colloid osmotic pressure of the blood and the hydrostatic pressure gradient derived from the difference in subarachnoid pressure and intracranial venous pressure (Fig. 1c, Model 1).

Hugh Davson noted in a review of the subject [52] that emerging physiological evidence that proteins could exit the SAS without restriction [53] argued against the presence of an osmotic gradient. For an osmotic gradient to be effective, membranes would have to be present that would hinder the outflow of proteins. However, if these membranes were intact, a buildup of proteins that are normally present in the CSF, such as albumin, would likely occur as no driving force would exist for their removal. This situation would be exacerbated during pathological situations such as meningitis and subarachnoid hemorrhage during which protein and cell content within the CSF are known to increase dramatically. In an experimental study in rabbits using intraventricular infusions of plasma or dextrans, Davson later concluded that the reduction of outflow observed by Weed may have instead been caused by an increased viscosity of the solutions or a possible blockage of the outflow pathways [54]. Therefore, in this case, the sole force driving outflow would be the difference between the hydrostatic pressures of CSF and dural sinus blood. This was consistent with physiological evidence from other investigators at the time that supported a concept of bulk outflow for CSF. For example, a direct relationship between SAS pressure and outflow has been since demonstrated by several groups [55–57]. In addition, different molecular weight particles exhibited similar dynamics of outflow [58, 59], which would also be consistent with a bulk flow mechanism.

An anatomical basis for unrestricted bulk outflow was suggested by the work of Welch and colleagues who described open channels within arachnoid villi in isolated segments of dural tissue in monkeys and dogs [60, 61]. These investigators used excised pieces of dura containing villi and the superior sagittal sinus that they set up as an occluding membrane between two tubes. They then altered the pressure and flow of fluids containing different size particles through the villi. A flow from the SAS side to the venous sinus side occurred at a specific opening pressure as a hydrostatic gradient was established. An increased flow occurred when the perfusion pressure was increased and the villi became distended. When the gradient was reversed, flow did not occur and the villi appeared collapsed (Fig. 1c, Model 2). This suggested to the authors that a valve mechanism must exist within the villi, similar to that originally proposed at the beginning of the century by Cushing [32]. Evaluation of the size of the particles that were able to be transported indicated that molecules up to the size of red blood cells (7.5 μm) could flow through. Examining the villi histologically, the authors described a circuitous network of tubes lined with “flattened mesothelial cells” within the villi that are in open communication with the lumina of the lacunae laterales [60]. These channels were later proposed to be endothelial cell lined spaces [62–64]. One must note that the perfusion studies were all performed post mortem on isolated tissues that might exhibit degradation over time. Therefore, it is not unlikely that pathways that are normally closed in the in vivo state were available for flow [26, 65]. Other authors suggested that apparent openings on the surface of the villi or granulations had been misinterpreted and were actually tortuous infoldings of the arachnoid tissue, which present with an undulating surface on tissue sections [27, 66].

The story was further complicated by anatomical evidence from electron microscopy that showed that the arachnoid villi did not actually completely penetrate through the endothelial lining of the dural sinus which indicated that a barrier in the form of tight junctions between the endothelial cells was present [67–69]. These researchers found that this non-fenestrated layer of endothelial cells was present even under conditions of elevated intracranial pressure despite an obvious distension of the villi itself. The authors concluded that an active process, such as micropinocytosis, must be necessary to transport proteins and other high molecular weight substances from the CSF (Fig. 1c, Model 3). However, such a mechanism is again at odds with the physiological studies that demonstrated relatively fast and unrestricted bulk flow of macromolecules from the SAS to blood [70]. Other authors also demonstrated that transport could occur after death arguing against an active transport mechanism [71, 72].

A potential compromise between these open and closed models of CSF outflow was suggested by Tripathi and Tripathi [73]. Extending upon work that was performed on the mechanism of aqueous humor outflow at the canal of Schlemm in the eye, it was found in primates using electron microscopy that a system of vacuoles within the covering cells of the arachnoid villus could transport solutes from the CSF space to the venous sinuses. It was proposed that these vacuoles are dynamic in nature and would occasionally form a continuous channel from the basal to the apical side allowing bulk flow to occur (Fig. 1c, Model 3). Another potential mechanism was speculated to be interendothelial gaps that may open to flow under favorable pressure gradients [27, 74, 75]. However, although Shabo and Brightman had also observed ‘narrow clefts' between endothelial cells, when they perfused horseradish peroxidase (HRP) into the SAS they could not detect that any reaction product had crossed the villus endothelium [76].

Andres [77] evaluated arachnoid villi of cats and dogs and found that there was no difference between the composition of the arachnoid layer at the villus and elsewhere in the skull. This work was confirmed by Krisch in rats and primates [78], who further criticized several earlier studies demonstrating an open communication between the venous sinus wall and the SAS [63, 79, 80]. She suggested based on an examination of the published electron microscopic images that cells identified as endothelial often lacked a basement membrane and that preservation artifacts may have led to a loss of intact arachnoid cells. In the anatomical preparations of Krisch, protusions into the dural sinuses appeared to be comprised of intact leptomeningeal layers [78]. If true, this would indicate that the barrier properties of arachnoid cell layer are present even at the regions covering the villus. Examination of human tissue has given further evidence to the concept of a continuous arachnoid cell layer at the cap of the arachnoid villi [29, 81, 82]. Thus, the original description of Key and Retzius of “two thin membranes”, in this case those of the arachnoid and the endothelium (Fig. 1c, Model 4), intervening between the SAS and venous lumen may ultimately be proven to be correct [2, 26].

In the end, we must reach a conclusion that anatomical studies have yet failed to demonstrate structures extending to the dural sinuses that would correlate with the observed physiological observations of unrestricted CSF outflow. What then would be the role of arachnoid villi or granulations? Some authors have suggested that they may act as pressure valves that open to flow under increased intracranial pressure [18, 78, 83]. Krisch proposed a rapid “volume buffering” function for arachnoid projections [78]. As the internal framework of the granulations is continuous with the SAS, under conditions of elevated intracranial pressure, the structures will first distend and eventually pathways for flow through both the arachnoid and endothelium into the venous sinuses could develop. Experimental evidence for an expansion of the villus under elevated pressure conditions exists for many species [60, 74, 75, 84, 85]. For example, Gomez and colleagues investigated the effects of pressure on arachnoid villi morphology in monkeys [74]. As the pressure gradient was increased from 0 to 250 mmH_2_O the arachnoid cells and fibers within the villi were more separated, opening more channels for flow within the villi. The endothelial cells flattened and gaps developed at the cell–cell junctions which became larger as the pressure gradient increased. Levine and colleagues, also working in primates, investigated the vacuoles within the endothelial cells under several different pressure gradients and found that the number of vacuoles and their size was dramatically increased at higher pressures [79]. Although some physiological evidence for a pressure valve function for arachnoid projections does exist [86], further studies in large animal models would be necessary to elucidate the anatomical mechanisms for outflow.

## Outflow along cranial nerves

### Olfactory route through the cribriform plate

The cribriform plate is the horizontal portion of the ethmoid bone. It derives its name from the Latin words *cribrum,* meaning sieve, and *forma,* meaning figure, in reference to the large number of foramina that allow fila olfactoria to extend from the olfactory bulbs to the nasal epithelium. An outflow of CSF through the cribriform plate into the nasal cavity is in fact the earliest pathway to be described. It dates back to the Roman physician Galen, who described that purification of “animal spirits” within the brain takes place through funnel-like structures in the plate [25]. Thomas Willis, in his work Cerebri Anatomii (1664), also suggested that fluid within the skull would drain out through the cribriform plate, however, this viewpoint was gradually lost to history [87, 88].

**Fig. 2:**
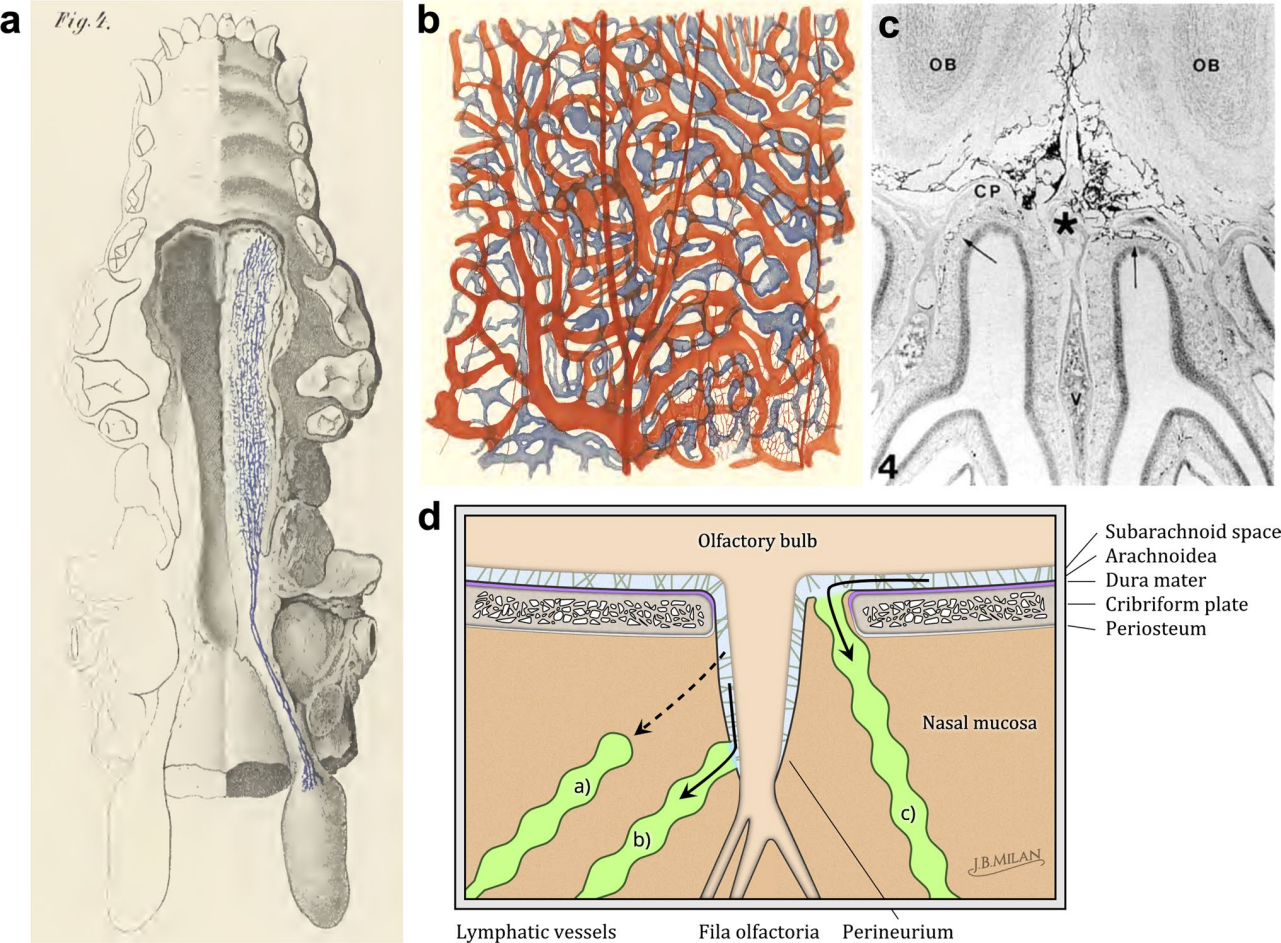
CSF outflow through the cribriform plate to nasal lymphatics.** a** Reproduction of an image from Key and Retzius [2] showing the lower surface of a dog's skull in which the lower maxilla and palate have been removed on one side to show the lymphatic vessels of the nasal and palatal submucosa filled with Richardson's blue dye from the SAS. The dye is transported to the deep cervical lymph glands of the neck. **b** Also from Key and Retzius [2], demonstrating Richardson's blue dye-filled lymphatics in the nasal submucosa of a rabbit injected into the “subdural” space of the brain. The authors noted that similar filling of vessels was observed in animals after injections into the SAS. **c** Reproduction of an image from Kida et al. [116] showing a decalcified coronal section from a rat that had been injected with India ink. Connections through the cribriform plate are evident with India ink particles found within lymphatic vessels on the nasal submucosa side of the plate (arrows). **d** Models for anatomical routes of CSF outflow to reach lymphatic vessels in the nasal tissue. Experimental support exists for three anatomical routes for tracers to cross the cribriform plate alongside the olfactory nerves. Model (**a**) represents an outflow pathway along a contiguous subarachnoid–perineural space (light blue). In this model, the perineurium is a continuation of the arachnoid mater, however, it does not form a barrier outside of the skull or is only loosely adhered to the nerve. Fluid and solutes have unrestricted access to the interstitium of the nasal (sub)mucosa (brown) where they then enter the lymphatic vasculature within this tissue (yellow). In model (**b**), CSF also crosses the plate through a perineural space but then has a direct pathway to lymphatic capillaries that surround the nerve in a collar-like fashion. Model (**c**) shows bona fide lymphatic vessels crossing the cribriform plate to directly access the CSF on the CNS side. In this model the arachnoid mater would not form a barrier in the region covering the cribriform plate. Scheme by Joachim Birch Milan

The first tracer experiment that revealed that there was an anatomical connection from the SAS to the nasal submucosal lymphatics was that of Schwalbe in 1869 [89]. Key and Retzius also demonstrated impressive filling of the nasal submucosal lymphatic vessels and deep cervical lymph nodes after injections of Richardson's blue dye in rabbits and dogs, although the previously mentioned criticism regarding high pressure injections in the post mortem state must be again noted (Fig. 2a,b) [2]. Interestingly, Lewis Weed also revealed this pathway in his well-controlled studies with dogs, although he regarded the lymphatic route in general as a minor or accessory pathway of CSF outflow compared with arachnoid villi [3]. Mortensen and Sullivan, working in vivo in dogs, were able to trace a pathway through the plate to the nasal mucosa and then within lymphatic trunks along the pharynx to the deep cervical lymph nodes [90]. In addition to these early studies, several dozen reports in a wide variety of species have been published that have confirmed that tracers injected into the CSF will exit across the cribriform plate, as exemplified in Fig. 2c [36, 37, 39, 47, 53, 56, 65, 72, 83, 91–136].

Investigators have suggested at least three possible anatomical pathways that may exist for CSF to penetrate through this structure (Fig. 2d):

1. Perineural pathways along olfactory nerve sheaths to reach lymphatic vessels of the submucosa through intervening interstitial tissue
2. Perineural pathways along olfactory nerve sheaths to directly reach lymphatic vessels of the submucosa
3. Through lymphatic vessels crossing the plate that might directly access the SAS

The bulk of the evidence has supported the existence of the perineural pathway associated with the olfactory nerves (fila olfactoria). This pathway is described as an extension of the meningeal layers such that the SAS is continuous with the perineural space along the nerves as they exit through foramina within the cribriform plate [47, 123, 137]. The dura layer has been described to blend into the periosteum of the lamina cribrosa, with the arachnoid layer continuous with the perineurium [47, 112]. The epineural covering of the nerve serves as the inner boundary of this sheath. The perineurium gradually diminishes in size as the olfactory filaments become smaller as they extend into the submucosa, becoming one cell layer thick at the superficial submucosal layer [112]. Two models were originally proposed for how fluid could escape from these perineural spaces to reach lymphatic vessels of the nasal submucosa [137, 138].

The first model suggests that the perineural cells are loosely adhered to the olfactory axon which allows fluid and solutes to easily access the interstitial spaces of the submucosa [139]. In this model, the perineurium offers no barrier to solute and fluid flow from the perineural space to the interstitium. Additionally, no real barrier would exist for entry of solutes into initial lymphatic vessels within the interstitial tissue of the nasal submucosa, as large clefts between lymphatic endothelial cells have been observed [111], similar to other regions in the body [19, 140].

In the second model the perineurium would exhibit barrier properties similar to the meninges within the skull [137]. This model would require cell mediated transfer of solutes and fluid or vacuolar mechanisms similar to those proposed above for arachnoid villi in order to reach the interstitial spaces of the nasal submucosa. This is unlikely, as the same objections could then be raised in reference to the many physiological studies demonstrating rapid bulk CSF outflow of macromolecules. However, one potential anatomical arrangement that could allow for an uninterrupted flow of CSF would be if the lymphatics directly adjoined to the perineural space, as proposed by Hoffman and Thiel [30] and by the more recent work of the group of Miles Johnston [83, 124]. In this version of the model, a “collar” of lymphatics surrounding the olfactory nerve rootlet isolates the perineural space from the surrounding submucosal interstitial tissue. This anatomical arrangement, if truly present, would have no known parallel elsewhere in the body. Within other fluid-filled tissue spaces, such as the peritoneal cavity, stomata normally are present in the mesothelial lining to allow direct drainage of fluid and solutes to lymphatic vessels [19, 141, 142].

Tracer studies have provided support for both of these models. Lewis Weed’s studies with Prussian blue precipitate indicated that the connections were not direct, as tissue spaces always intervened between the perineural spaces and lymphatics. Several other studies performed in rabbits are in agreement [47, 91, 92, 108]. Bradbury and Cserr summarized after injections of FITC-dextran or Evans blue: “In the case of the olfactory nerves, there was massive coloration of the outside of the olfactory bulbs, of the surrounding dura, of both sides of the cribriform plate and of the olfactory epithelium and its submucosa. The fluorescence or staining of the olfactory epithelium extended forwards and laterally under much of the nasal mucosa and even into the air sinuses” [22]. Two ultrastructural studies using HRP and ferritin, respectively, demonstrated that these tracers escaped from the loose connective sheaths of the perineural spaces to reach the interstitial space [111, 112]. On the other hand, tracer studies from the group of Miles Johnston have supported the more direct route from the perineural spaces to lymphatic vessels surrounding the nerve rootlets [83]. In several species, Microfil was injected post mortem and demonstrated filling of lymphatic vessels from the perineural space with very little evidence of the contrast agent in the connective tissue interstitium [124]. Although such a direct pathway is intriguing, it is important to indicate that high pressure injections with a viscous agent in the post mortem state were utilized to visualize this pathway. It may be possible that the large particles of Microfil entered and filled the lymphatics as a path of least resistance rather than spreading through the interstitial spaces of the submucosa.

A third emerging model is based on evidence from rodents showing that lymphatic vessels appear to cross through the cribriform plate which may allow them to access the SAS directly [116, 133, 134, 143–145]. This would offer an alternate pathway, one not necessarily associated with the olfactory nerves, for direct access of CSF and its constituents to the lymphatic vessels. Anatomical support for this pathway is found from developmental studies in rats that have indicated that the arachnoid barrier layer of the meninges is not present above the cribriform plate [146] and thus would not accompany the olfactory nerves outside of the skull. Whether the arachnoid barrier layer is completely absent at the cribriform plate or a porous membrane remains under debate. According to Walter et al.: “At the place where the fila olfactoria enter the cribriform plate, the subarachnoid space formed a funnel- or pocket-like space around the fila olfactoria. Although the surface of this space was covered with arachnoid cells, partial interruptions were occasionally observed in this layer” [123]. As the dura does not appear to be present at this location either, it is not clear whether the lymphatic vessels crossing to the CNS side of the cribriform plate can be characterized as meningeal or dural lymphatic vessels, as defined in the next section. Evidence for this direct pathway does not yet appear to exist in species besides rodents. Faber, working in rabbits, found that no lymphatic vessels could be demonstrated through the cribriform plate in serial sections [47]. Lowhagen et al. were also unable to confirm a direct lymphatic pathway in human post mortem specimens [117].

Some older studies utilizing injections post mortem have suggested pathways through the plate not associated with perineural spaces or lymphatic vessels [2, 94]. Later investigators have considered that the pressure used in injecting tracers within the cranial space may have caused artifacts that have ruptured existing barriers. Faber found Prussian blue pigmentation located within the nasal cavity, but he was unable to elucidate how the tracer passed through the nasal mucosal epithelium [47]. Several investigators, including Faber, have also shown that retrograde pathways also exist from the nasal cavity to the SAS or potentially directly to the brain [47, 147–149]. These pathways were implicated in the spread of viruses to the CNS and have recently been explored as a potential delivery route for pharmaceuticals or stem cells.

Thus, there is very strong evidence for an efflux route for CSF through the cribriform plate in many different species, although the detailed anatomical connections from the SAS to lymphatic vessels still need elucidation.

### Optic route

Routes along other cranial nerves may also serve as additional outflow pathways. Of these, the best described are the optic nerves. The optic nerve is contained with a double sheath of meningeal tissue, with an outer fibrous layer of dura and arachnoid tissue and an inner layer of pia mater covering the nerve [150]. Magendie, who first coined the term cerebrospinal fluid, even provided a description that the CSF contained with the SAS of the optic nerve reaches the orbit [151]. Schwalbe demonstrated experimentally in 1869 (translated from the original German): “The injection fluid flows from the subarachnoid space by the optic canal into the orbit and first fills the space between the inner and outer optic nerve sheath which thus turns out to be a continuation of subarachnoid space; the mass then simultaneously penetrates into a space which is located between the optic nerve and the *retractor bulbi* muscle” [89]. This finding too has been confirmed several times in multiple species [2, 3, 36, 37, 39, 40, 53, 56, 65, 72, 92, 95, 96, 98,99, 101–104, 106, 109, 116, 118, 121, 122, 127, 128, 130, 135, 152–157].

The route from the distal end of the optic nerve sheath to the connective tissue surrounding the eye has been examined histologically and/or at an ultrastructural level in several species. In rabbits, Brierley and Field found India ink particles passing through the dura-arachnoid sheath of the nerve at a location close to the sclera. The particles entered the adjacent fatty tissue. These authors were unable to confirm the presence of endothelial-lined lymphatic vessels within the orbit and showed that the spread of India ink particles “took place along more or less definite lines through the fat” [152]. De la Motte considered the arachnoid layer continuous throughout the length of the optic nerve, similar to the closed model that had been proposed at the olfactory nerve, and that phagocytosis and vesicular transport must be responsible for the presence of fluorescent dextran and horseradish peroxidase that was observed in the surrounding tissue [106]. Shen et al. demonstrated in the hamster that the continuation of the SAS within the optic nerve extends to the lamina cribrosa sclerae [154]. Unlike the olfactory nerves, both the arachnoid and dura tissue have been described to accompany the nerve out of the skull and together blend into the outer sclera layer. At this “terminal portion” of the nerve, ferritin particles could be seen to enter tortuous intercellular channels that passed through an arachnoid trabecular meshwork to reach the posterior intraorbital connective tissue. The authors concluded that the arachnoid barrier layer at the termination of the SAS of the optic nerve is not continuous. These same authors confirmed their findings in rabbits but in neither species could they demonstrate the pathway to lymphatic vessels. Kida et al. found that India ink particles filled the SAS to the orbit but did not enter the surrounding tissue, indicating that a size exclusion might be present in the microchannel pathways [116]. Ludemann et al. observed in cats that an outflow of tracers at the distal terminal portion occurred [122]. They found numerous excavations in the SAS that breached the outer arachnoid layer to reach the neurothelial cells lining the dural–scleral interface. Pores were found in this neurothelial cell layer. Contrast agent was found in the conjunctival lymphatics, but no other lymphatics could be demonstrated within the orbital tissue. In mice, lymphatics have been observed along the dural nerve sheath of the optic nerve and in the fascia of the optic muscles [143]. It is unclear at this time if either of these would represent a route for CSF from the SAS of the optic nerve to the conjunctival lymphatics.

There has been some debate as to the relative importance of the optic nerve route compared with the olfactory route. Although Bradbury and Cole could confirm outflow of fluorescent dyes along the optic nerve to the conjunctival tissue, they found that when they injected tracers directly into this space they could not recover the tracers within the deep cervical lymph and concluded that this route was not nearly as important as the nasal route [153]. However, it has been found that the route from the orbit appears to be through a superficial lymphatic route rather than towards the deep cervical lymph nodes. From the connective tissue of the orbit the lymphatic vessels track alongside the angular and facial veins to reach collecting vessels leading to (sub)mandibular or superficial cervical lymph nodes [98, 130]. In mice the route along optic nerves appears to be a significant contributor to the CSF outflow, as tracer signals were found in orbit-draining collecting vessels within 10 min of lateral ventricle infusion and signals within the superficial cervical (mandibular) lymph nodes reached levels that were comparable to those found in deep cervical lymph nodes [158]. However, as some of the afferent vessels leading to the superficial cervical lymph nodes appear to originate from other sources, including the nasal cavity [130], it is impossible to make any direct proportional comparison between outflow routes.

### Other cranial nerves

The evidence for CSF outflow along other cranial nerves is not yet conclusive. The trigeminal nerve (CN V) has been implicated in several studies [40, 65, 96, 99, 101, 159, 130]. However, others have not seen transport of tracers past the trigeminal ganglion [22, 95]. As with the nasal pathway, the trigeminal nerve has been implicated for retrograde drug delivery to the CNS indicating that communication can be induced between the periphery and the SAS [149]. A similar story present itself with the facial nerve (CN VII) with some investigators unable to demonstrate tracer outflow further than the geniculate ganglion [95], yet others have described an outflow of CSF tracers reaching lymphatic vessels near the stylomastoid foramina [104, 130, 160]. Lymphatics have also been identified within the perineural sheaths of the facial nerves in mice [141].

Many investigators have demonstrated that the SAS and perilymph of the ear are connected through the perineural space of the acoustic nerve (CN VIII) [2, 3, 89, 95, 103, 104, 161]. From the perilymph of the scala tympani, Arnold et al. has traced India ink particles to the lymphatic vessels of the middle ear mucous membrane in guinea pigs [104]. Manzo et al. were able to trace HRP to lymphatics in a similar manner in rabbits [161]. The quantitative importance of this pathway to total CSF outflow remains to be elucidated, as other investigators have failed to demonstrate CSF-infused tracers reaching lymphatic vessels at this anatomical location [22, 118].

More solid evidence exists for CSF outflow at the jugular foramina [45, 65, 89, 92, 104, 130]. Three cranial nerves exit the skull at this location: the glossopharyngeal (CN IX), vagus (CN X), and accessory (CN XI) nerves. Our group has visualized tracer leaving along the nerves exiting the jugular foramen with connections to lymphatic vessels leading the deep cervical lymph nodes [130]. This site may also be an important CSF egress location through meningeal (dural) lymphatic vessels [160], as described in the next section.

## Meningeal (dural) lymphatic vessels

Reawakened interest in the pathways of CSF outflow is largely due to the (re)discovery of lymphatic vessels in the meninges of the CNS by two studies published in 2015 [144, 162]. Both studies described a network of bona fide lymphatic vessels in the dura mater, the outermost of the three layers of meninges. These vessels were found in close proximity to the superior sagittal and transverse sinuses of the dorsal dura and, more extensively, along blood vessel networks of the basal skull [144]. Interestingly, the vessels of the dorsal region lack lymphatic valves, while sporadic valves were found in the vessels of the base. Since these studies, these vessels have also been shown to exist in both non-human primates and humans [145, 163, 164]. Although these vessels are often referred to as “meningeal lymphatic vessels”, or even “brain lymphatics”, these terms may lead to misinterpretation if these vessels are solely confined to the dura mater, which technically lays outside the barriers of the CNS.

**Fig. 3:**
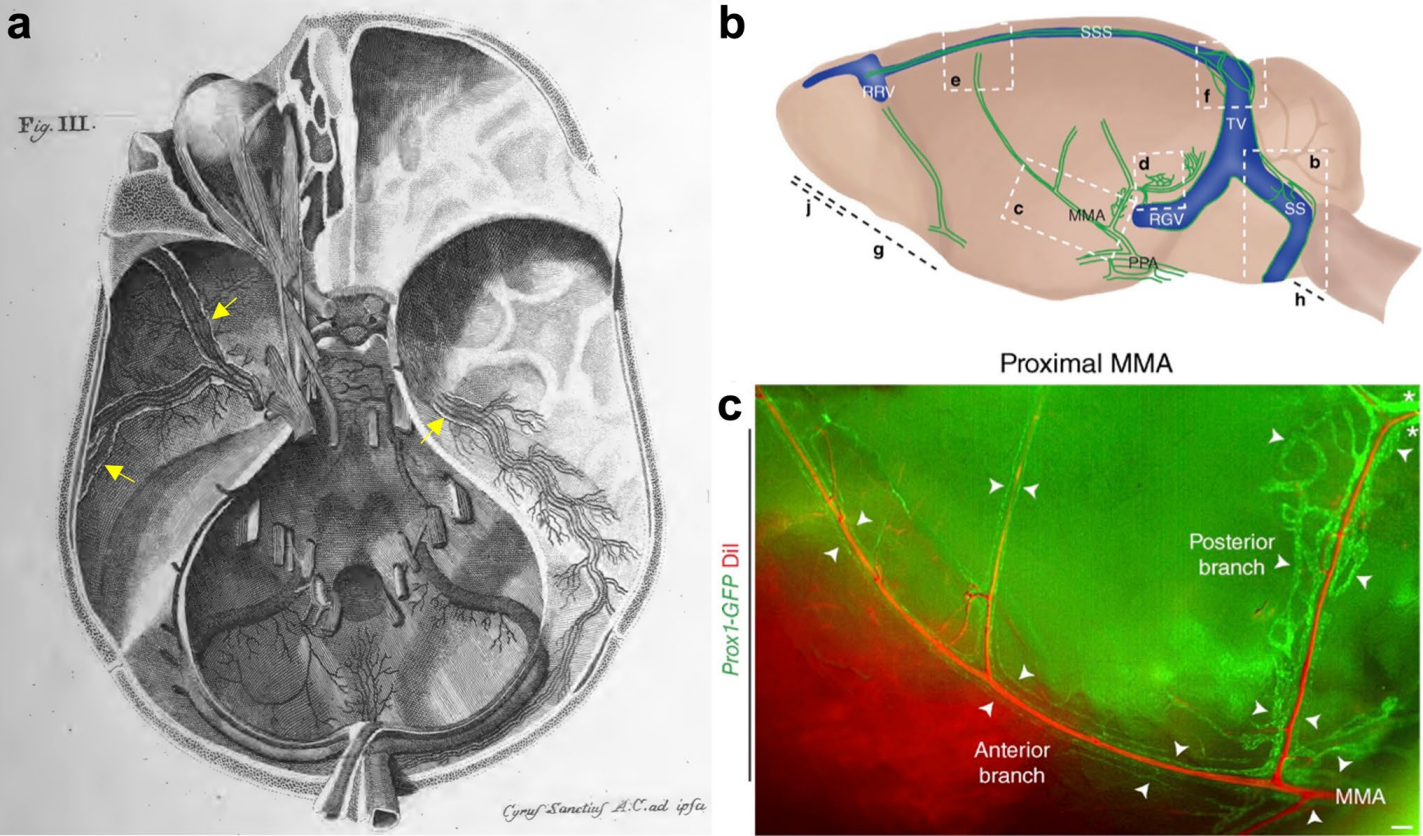
Dural lymphatic vessels of the basal skull in mice and humans. **a** Reproduction of a plate from Mascagni, 1787 [165]. Using injections of colored beeswax and mercury in human cadavers, dural lymphatic vessels (lighter colored vessels indicated by yellow arrows) were identified at the base of the skull alongside the middle meningeal arteries and veins and were found to leave the skull with these vessels at the foramina spinosa. **b** Schematic from Aspelund et al. [144] demonstrating regions of the dura found to contain lymphatic vessels in the Prox1-GFP transgenic reporter mouse. *MMA* middle meningeal artery, *PPA* pterygopalatine artery, *RGV* retroglenoid vein, *RRV* rostral rhinal vein, *SS* sigmoid sinus, *SSS* superior sagittal sinus, *TV* transverse vein. **c** Close up of the region c shown in panel b, indicating the proximity of the Prox1-GFP lymphatic vessels (green) to the branches of middle meningeal arterial (MMA) vessel network [perfused via intravenous DiI dye injection (red)] in an analagous situation to that described by Mascagni in humans

Despite the great amount of attention in the scientific community that the rediscovery of these vessels has received, it is important to note that several investigators have identified or acknowledged the presence of these vessels in the past [116, 159, 165–173]. Of note, Paolo Mascagni in 1787 is deemed to be the first to describe dura mater lymphatics and his images of these vessels in the base of the skull of humans are strikingly similar to the network shown in Aspelund and colleagues in mouse (Fig. 3) [144, 165, 174]. Lymphatics within the dura mater attached to the skull at the jugular foramen have also been previously identified [170]. Of particular importance is the work of Andres in rats, who visualized dural lymphatic vessels along the wall of the superior sagittal sinus and in close vicinity to the blood vessels of the dura mater [173]. It was described that these lymphatic vessels left the skull at the cribriform plate, as well as alongside the transverse sinus or together with the middle meningeal artery.

One significant objection that had been repeatedly raised in the past to these vessels playing a significant role in CSF outflow is the existence of the arachnoid barrier layer between the SAS and interstitial tissue of the dura mater [159, 171, 172]. Early investigators using trypan blue dye demonstrated a clear compartmentalization between the dura and the CNS proper, with the dura staining blue only after intravenous injections and leptomeninges staining blue only after intrathecal injections [39, 175]. The arachnoid barrier was examined on an ultrastructural level in many species by Nabeshima et al. and was described as comprised of a few layers of closely apposed, epithelial-like cells connected by extended complexes of tight junctions [176]. The arachnoid barrier essentially separates the CNS from the dura mater, which is supplied by a blood vessel network with properties similar to those in peripheral tissue. Thus, one might imagine that the dura mater lymphatic vessels primarily exist to collect extravasated fluid and solutes within this specific tissue, similar to most tissue beds in the body. This concept is supported by MRI studies in humans and primates demonstrating uptake of small molecular weight contrast agents into the dural lymphatic vessels after intravenous injection [163]. Brochner et al. found that the arachnoid barrier layer could be identified using the junctional protein claudin-11 and performed a series of staining in developing rats and in human fetal tissue [177]. They found that the barrier cell layer was intact around the brain, was particularly prominent at the base of the skull and demonstrated interruptions only where cranial nerves penetrated the dura-arachnoid layers. This is consistent with earlier work using electron microscopy of tissue from cats and dogs from Andres [178].

Studies utilizing CSF-injected tracers to evaluate potential pathways through the arachnoid barrier or probe the function of dural lymphatic vessels have provided inconsistent results. Krisch et al. found that the interstitial space of the dura mater was functionally separated from the CNS by intercellular clefts filled with electron dense, granular material [159]. These authors could not detect HRP particles to cross this barrier in either direction, either from the dura mater interstitium to the SAS after leaking from the systemic blood circulation or from the SAS to the dura mater after lateral ventricle injection. They were able to observe clear filling of dural lymphatic vessels only after injection of HRP directly into the outer meningeal layer. They thus concluded that these vessels drained the “hemal mileau” of the dura. Walter et al. similarly found that inner arachnoidal cells covering the dura mater in the region of the olfactory lobes “often took a stellate shape and formed a cellular mesh on the dense connective tissue of the dura mater” [123]. Despite numerous India ink particles located within phagocytic cells of this mesh, these authors could not detect particles in the dura mater. Butler et al. injected HRP under several different pressure conditions and found that tracers reached the dural lymphatics over the cerebral convexities in cats only under conditions of high pressure (> 200 mm H_2_O) [172].

In contrary, Louveau et al., using a thinned skull preparation in mice was able to demonstrate uptake of Evans blue dye and quantum dots into dura mater lymphatic vessels near the superior sagittal sinus after cisterna magna injection [162]. This same group later found that cisternally injected fluorescence-labeled antibodies specific for the lymphatic marker LYVE-1 were able to access dural lymphatic vessels in subsequent whole mount preparations [179]. They postulated the existence of “hot spots” at particular locations, such as along the transverse sinus, at which the vessels might have direct access to the SAS. These potential access points through the arachnoid barrier have yet to be assessed on an anatomical level.

Our own group has been unable to confirm uptake of several different CSF-injected tracers into the dorsal lymphatic network in mice [130], a result that was recently replicated by another study [160]. In contrast, in collaboration with the group of Kari Alitalo at the University of Helsinki, we found that pegylated dyes had some access to the basal dural lymphatic network, at least after injection into the brain parenchyma [144]. Ahn et al. also found uptake of quantum dots after cisterna magna injection into dural lymphatic vessels at the skull base with exit of tracers at the petrosquamous fissure and the jugular and stylomastoid foramina [160]. Using immunostainings for the junctional protein E-cadherin it was suggested that the arachnoid barrier layer may not be intact at the locations in the base of the skull where the tracer accesses the lymphatic vessels. These authors concluded that uptake by basal dural lymphatics was the main route for macromolecular uptake and clearance of CSF. However, this study did not evaluate outflow routes through the cribriform plate or other cranial nerve locations.

Other authors have described lymphatic vessels in the dura mater of the optic nerves [143, 180]. Killer et al. described lymphatics on optic nerve sheath of humans and found that tracer could penetrate the arachnoid layer when India ink was injected directly into the SAS of the optic nerve post mortem. More tracer was found within dural lymphatics at the bulbar end of the optic nerve. Brinker et al. determined that under normal conditions the arachnoid barrier layer is intact until the extracranial distal portion of the nerve where the channels previously described by Erlich et al. are present [181, 156]. At high injection pressure, they described the arachnoid cells becoming flattened, an expansion of the extracellular space and the development of intracellular and intercellular pores. These pores were representative of a mechanically disrupted meningeal fluid barrier, as evident by passage into the orbit of X-ray medium or erythrocytes from autologous blood that had been injected into the cisterna magna.

In sum, lymphatic vessels have been localized in the dural meningeal layer in several different locations within the skull. At this time, it still remains to be determined how exactly and under which conditions these vessels participate in the drainage of CSF fluid and solutes across the arachnoid barrier layer.

## Outflow from the spine

When compared with outflow from the cranium, the mechanisms for outflow of CSF from the spine have been less studied. Most investigators have reached agreement that outflow from the spine, regardless of whether it occurs through venous or lymphatic routes, is much less substantial than from the cranial region under normal conditions [3, 45, 182–184]. Much attention has been focused at the so-called “subarachnoid angle” where the arachnoid membrane forms a cul-de-sac at the site of the emerging spinal nerve roots [185]. This extension of the SAS is particularly prominent along dorsal nerve roots. At the cul-de-sac, loose tissue containing basement membrane is present between the SAS and the endoneurium that merges into the dorsal root ganglion. Researchers have long debated if and how CSF would clear from this space under normal physiological conditions. Some investigators believe arachnoid villi are present in this area and that these would connect to the venous plexus that surrounds the nerve root [186] or, alternatively, to epidural tissue and lymphatics [100]. Still others believe that a continuous subarachnoid–perineural space extends along the nerves for some distance even beyond the spinal ganglion [187, 188]. More recent evidence has supported potential pathways through the arachnoid layer at this anatomical location to lymphatics in the spinal dura mater or epidural tissue [145, 189, 190].

Weed concluded in his initial studies that outflow from the spine must be lymphatic in nature as he was unable to find arachnoid villi in this region [3]. However, working in the same laboratory, Elman later reported the existence of arachnoid villi in the spine [191]. At the site of the subarachnoid angle, accumulations of arachnoid tissue were found that permitted passage of the Prussian blue substrates through the dura to the tissue surrounding the nerve roots of the spinal cord. These arachnoid villi were not shown histologically to project directly into veins until work in newborn rats from Woollam and Millen [192]. Carbon granules that had been injected daily into the cisterna magna from birth until three weeks were found within the connective tissue of the villus but not in the veins itself. The authors interpretation was that the colloidal carbon was carried in the normal outflow of CSF towards veins but was unable to penetrate the endothelial lining of the venous sinus, leaving deposits within the core of the villi. Interestingly, carbon granules were also found within the lymphatic vessels leading towards lymph nodes on the dorsal abdominal wall. Welch and Pollay working in serial sections of tissue from two species of primates found arachnoid villi near the lumbar nerve roots [184]. However, only in 5 out of the 32 nerve roots studied were arachnoid villi directly associated with dural veins.

Kido et al., in human cadaver tissue, identified three types of arachnoid villi or granulations in the spine [193]. The first was localized internal to the nerve root dura mater, the second (and most common) reached into the dura mater and the third extended through the dura completely to the epidural space. Dye that had been perfused into the spinal SAS in the cadavers was found to some cases within the lumen of venous sinuses, confirming earlier results from this group demonstrating CSF outflow of radiopaque contrast material during post mortem infusions in dogs [72]. In another study from the same group, Gomez et al. found that “at the regions that the pia and arachnoid fused in an irregular manner, prominent proliferations of arachnoid cells were frequently seen” [194]. Structures that gave the appearance of arachnoid villi or granulations were found in a total of 51 nerve roots in three dogs and 48 nerve roots in three sheep. Prussian blue that had been injected into the spinal SAS shortly after death were found in spaces between the arachnoid cells and, occasionally, the cells were separated from veins only by a single layer of endothelium. However, an anatomical pathway for CSF flow through this endothelial cell layer, and thus through spinal arachnoid villi, has yet to be elucidated, similar to the case within the cranium.

Several authors have demonstrated that a lymphatic outflow of tracers can occur at spinal nerve roots. Early investigators such as Quincke and Key and Retzius demonstrated that injected tracers were found to extend along the nerves for some distance from the spine [2, 40], which they took to indicate that a perineural pathway was open to carry CSF beyond the subarachnoid angle, a viewpoint more recently espoused by Bechter [188]. However, other groups did not observe India ink further than the intervertebral (dorsal root) ganglion when injection pressures were well-controlled [95, 195]. Accumulations of India ink remained confined at the cul-de-sac of arachnoid tissue at this location which was referred to as “nerf de congestion” by the French authors and “Tuschenmattchen” by the authors writing in German. Brierley and Field, writing later in English, described these structures as “ink cuffs” [100].

In the lab of Speransky, an extensive series of experiments were performed using India ink injected in dogs to elucidate CSF outflow pathways to the lymphatic system [45]. These investigators had succeeded in establishing a method through which the cranial and spinal subarachnoid spaces were separated from each other in living dogs using rings of muscle tissue wrapped around the spinal cord [95]. It was only possible to fill the lymph nodes along the abdominal wall when introducing the ink into the caudal region of the isolated spinal space. Ink introduced in small volumes in the intact SAS either into the cisterna magna or at the caudal spine did not demonstrate significant outflow from the spine [168]. Two rather illuminating studies on spinal outflow were performed in this lab by Galkin [97, 196]. In one, tissue overlying the cribriform plate was separated from the lamina cribrosa, rupturing the fila olfactoria. The foramina in the cribriform plate subsequently became sealed by connective tissue. Under these conditions, injections into the caudal SAS exhibited a filling of the abdominal lymph nodes similar to that seen when the cerebral SAS was isolated. Interestingly, the deep cervical lymph nodes remained unstained indicating that these are filled almost exclusively through the nasal route under normal circumstances [97]. The second study by Galkin utilized two rings of muscle tissue on the spinal cord to isolate three separate regions of the SAS: defined as upper, middle and lower regions. Injections performed into each of these regions demonstrated that lymphatic outflow occurred only from the cervical (through the nasal route) and lumbo-sacral regions, with essentially no outflow found from the thoracic region of the spine [196].

Brierley and Field performed injections of India ink into the lateral ventricle of rabbits and evaluated the outflow to several potential draining lymph nodes at different time points [100]. Examination of the table published in their report indicates similar results to that seen by the group of Speransky. At early time points India ink is found in the deep cervical lymph nodes, followed by the superficial cervical and the sacral lymph nodes. Only at later time points or after multiple injections were India ink particles found in lymph nodes that could be draining the thoracic region. These authors evaluated the outflow locations at the “ink cuffs” and concluded that “particles pass most readily out of the spinal subarachnoid space from the cul-de-sac just proximal to the posterior root ganglion of each spinal nerve, especially in the region of the second and third cervical nerves and of the three large roots contributing to the formation of the sciatic nerve” [101]. My laboratory has been able to repeat this finding in mice by using pegylated near-infrared fluorescent tracer and transgenic Prox1-GFP lymphatic reporter mice. We were able to demonstrate that the outflow of tracers injected into the lateral ventricle occurs to lymphatic vessels predominantly at the sacral region of the spine [184]. The tracers then drained to the sacral and deep iliac lymph node chains to return to the blood via the thoracic duct. A primary outflow site at the caudal end of the spine would imply that a directional descent of CSF from its site of production within the ventricles would occur under normal conditions. Indeed, routes for this caudally directed flow within the spinal column were identified both within the central canal, confirming earlier work [197, 198], and in the spinal subarachnoid space towards the lumbar sac. In fact, a caudal spread of radiolabeled agents has been demonstrated in monkeys and humans using ventriculography [199, 200]. Although previous investigators have found tracers within thoracic or lumbar nodes [116, 157], it is important to note that lymph in efferent lymphatic vessels from the sacral and iliac nodes will traverse these nodes during its passage to the thoracic duct [184]. Therefore, evidence of tracer within these nodes does not necessarily imply direct lymphatic drainage from these regions of the spine.

Studies from several species have found dural or epidural lymphatic vessels to be in abundance in the spine, particularly in the cervical region [145, 168, 189, 190, 201]. However, one must assume that a functional arachnoid barrier is also present in these regions. Supporting this concept, Miura et al. was able to fill these vessels with India ink only under high injection pressure conditions [201]. Zenker et al. has examined, on an ultrastructural level, potential pathways through the arachnoid layer of the cervical spine in rats [189]. In the subarachnoid angle region (called the meningeal angle by these authors), the arachnoid layer is reduced in size to two or three layers thick and the dura mater is thus closely associated with the SAS. Tight junctions were not always found in this region. Ferritin particles which were infused in vivo into the cisterna magna were found to have penetrated the arachnoid membrane in an intercellular manner and reach the dura mater. Transcellular pathways were also identified through uptake by arachnoid-lining cells and transcytosis. Macrophages, which were in abundance in the subarachnoid angle, also appeared to take up ferritin. The ferritin was found within dural lymphatics, but also within thin-walled veins in the dura.

Thus, a consensus has emerged that there is CSF outflow from the spine along spinal nerve roots, with the strongest evidence supporting an outflow to lymphatic vessels from the dorsal aspect of the lumbo-sacral region.

## In vivo quantitative studies

Removal of CSF and its solutes from the ventricles and SAS is dependent upon bulk flow in addition to the passive diffusion and/or active transport of specific constituents. This means that the choice of tracer will have a dramatic effect on the experimental results and the interpretation of such. For assessment of the outflow pathways of CSF, inert tracers that will follow the bulk flow pathways are preferred, meaning careful attention should be given to the molecular weight, lipid solubility, surface charge and the existence of known transporters within the CNS to blood. For example, one early study injected methylene blue in the CSF and detected a rapid outflow of this dye to the torcular venous sinuses [41]. They concluded that CSF must quickly exit to venous blood. However, we now know that methylene blue can cross the blood–brain barrier (after intravenous administration) due to its lipid solubility that allows it to easily cross plasma membranes [202, 203]. Another early study used phenolsulfonphthalein, which was found by Dandy and Blackfan to be present in urine minutes after an injection into the cisterna magna [37]. Yet, it was later found that this substance has active transporters that facilitate its quick efflux into the blood vascular system [58, 204].

The introduction of radioactive isotopes to physiological studies in the 1940s provided a means of tracing the movements of naturally occurring ions or water in CSF. The results from these experiments appeared to show unexpected routes of absorption [205, 206]. Thus, in studies performed in humans by Sweet and co-workers [206, 207], radiosodium and deuterium-labeled water were administered intravenously, or into the ventricles or cisterna magna. By analyzing serial samples of CSF from a lateral ventricle, the cisterna magna and the lumbar sac, and from the blood, the authors suggested that formation and clearance of the fluid was occurring throughout the CNS. These conclusions were later criticized by Selverstone [208], a co-author of one of these earlier reports [206], who pointed out that these experiments had been misinterpreted, for an exchange of ions demonstrates only the permeability of a membrane and does not indicate that there is a flow of fluid into or out of the system. It was impossible to measure whether an unlabeled ion had exchanged in the opposite direction during the experiments. In fact, co-infusion of unlabeled isotonic NaI into the CSF will dramatically reduce the efflux of I-labeled Na from the SAS [209]. Strikingly, in this case, the radiolabeled sodium appears to spread within the SAS in a convective fashion similar to labeled albumin. More recent work using injected radiolabeled ions and water from the group of Bulat and Klarica, has also been interpreted to mean that CSF does not exhibit a directional flow through the CNS [210]. These authors have used these findings to support their claims that CSF is not produced by the choroid plexus, nor absorbed at specific anatomical sites (i.e., arachnoid villi or lymphatics). However, this work suffers from the same pitfalls as these earlier studies, i.e., the authors are measuring tracer exchange rather than bulk flow.

Several investigators have attempted to measure the direct outflow of CSF to blood by injecting labeled substances in the ventricles and recovering these tracers in the urine or blood. For example, Prockop, Schanker and Brodie injected inulin, sucrose and mannitol into the lateral ventricle of rabbits and compared the dynamics in urine with injections of the same dose of substances in the blood [58]. The dynamics of all three substances were similar with a delay of around 1 h before substances were detected in the urine after intraventricular injection, but all three were present almost immediately within the urine after intravenous injection. The highest molecular weight substance, inulin, was detected in higher quantity showing its utility as a bulk flow tracer. Data from Davson in rabbits appears to be a more convincing demonstration of direct tracer uptake to blood, as radiolabeled albumin was evident in the blood by 30 min (the first time point assessed) after the initial infusion. However, it is important to note that after a bolus infusion of 100 μL of the tracer, another 100 μL bolus of artificial CSF was made followed by a continuous infusion of 60 μL/min of artificial CSF in order to “enhance the flow […] through the cerebrospinal fluid compartment” [70]. This constant infusion most likely dramatically accelerated the outflow dynamics. Mann et al. demonstrated that both inulin and 0.5 μm polystyrene beads could be detected in sinus blood of rats within three minutes of the start of the infusion. However, the authors infused the substances at 22 μL per min into the lumbar SAS, a rate that quickly led to intracranial pressures of over 700 mm H_2_O, far exceeding normal levels [211].

The first to attempt a quantitative study to assess the proportion a macromolecular label draining by the lymphatic vessels was Courtice and Simmonds in 1951 [53]. These authors investigated the removal of Evans blue labeled blood plasma from the SAS of cats and rabbits. Through cannulations, the dye–protein concentration was estimated at several time points up to 24 h in CSF, circulating blood and cervical and thoracic duct lymph. By comparison of the recovered proportions in each of these compartments, it was concluded that the greater part of the dye–protein appeared to have reached the blood by a route other than the lymphatics. These experiments have had a profound influence on later thinking (being cited in high impact journals to this very day [212]) and have served as evidence to minimalize the role that lymphatics have in the removal of macromolecules from the SAS. It is interesting to note that the authors report that in some rabbits, no dye–protein absorption to blood was detected until after one hour, while dye–protein was found to have clearly traversed the meninges through the cribriform plate to enter the nasal lymphatics or through the optic nerve to reach the retroorbital tissue.

The group of Michel Földi, repeating this type of experiment in dogs [213], criticized Courtice and Simmonds on the grounds that it would be impossible to retrieve a significant portion of lymph from a cannulation of one major lymphatic trunk leading to the deep cervical lymph nodes. Using a different approach, these authors directly compared the radioactivity in cannulated lymph to samples of femoral arterial and jugular venous blood at several time points after introduction of radiolabeled albumin to the cisterna magna in dogs. They showed that the tracers were evident in the cervical lymphatic vessels before they were detected in either venous or arterial blood. It was also shown that the jugular venous signals were never above those in the arterial blood. To the authors, this was consistent with a model in which lymphatic vessels were primarily responsible for the outflow of CSF, although later this group appeared to temper this viewpoint and attribute to veins the greater role for removal of protein and fluid [214]. Földi also developed a complicated surgical technique in which ligation of the lymph vessels or removal of lymph nodes in the cervical region was performed in rats and dogs [215, 216]. This led to quite surprising results in which a “lymphostatic encephalopathy” developed that was associated with elevated ICP, cerebral edema and behavioral changes in the animals. Sadly, this pioneering work was paid little heed at the time.

In a series of well-controlled studies in rabbits, cats, and primates, the group of J. Gordon McComb evaluated the route of CSF drainage at different infusion rates of radiolabeled serum albumin into the lateral ventricle or cisterna magna [56, 109, 114]. Through surgically implanted cannulas, the authors sampled the blood at the superior sagittal sinus and at the femoral artery at several timepoints after the initiation of the infusion. As in the earlier study by Földi, if tracer was exhibiting efflux through arachnoid villi then one would expect that the concentrations in the blood at the superior sagittal sinus would be higher than the concentrations in the systemic blood. However, at no time point in all three species was this the case. Even at infusion rates four times higher than the initial rate, resulting in a significant increase in intracranial pressure, the authors could not find evidence of direct efflux of the tracer to blood in primates [114]. In addition, a delay was evident before signals were detected in the blood at either cannulation site. This initial time until detection in blood became shorter when greater infusion rates were used, supporting a bulk outflow mechanism. Sampling several tissues at the end of the experiment, the authors could find little evidence for significant transport of tracer to the dorsal surface of the cerebral convexities where arachnoid villi have been found to be most abundant. Instead, high concentrations of radiolabeled albumin were found at the base of the brain, olfactory bulbs, optic nerves and episcleral tissue and in deep cervical lymph nodes. Using a similar experimental approach, the Johnston group examined CSF outflow of radiolabeled albumin in neonatal lambs. This group could detect an enrichment of tracer concentrations in the superior sagittal sinus blood as compared to vena cava, albeit only at elevated intracranial pressures (~ 200 mm H_2_O) [120].

The group of Michael Bradbury published several studies in the early 1980s in which cannulations of cervical lymphatic vessels were made in an attempt to quantify the proportion of CSF draining through this route in rabbits and cats [22, 108, 153]. They estimated that, in the rabbit, 30% of the CSF tracer was draining within lymphatics towards the deep cervical lymph nodes, while in the cat the estimated amount was close to 13%. Their study did not take into account tracer that may have be transported by other lymphatic routes or beyond the time points that the lymph was recovered from the anesthetized animals. Thus, their values may be substantially underestimated. In fact, the authors were not able to account for up to 50% of the tracer in either species after subtracting for the amount present in blood or still within the CNS tissue at the end of the study, an observation also made by other groups [56, 70]. If there were direct outflow to blood from the SAS, one would not expect such high levels of tracer unaccounted for. Indeed, if phenolsulfonphthalein is injected into the cisterna magna, a substance identified to have transporters for efflux to blood, recoveries are described to be 100% [58]. This discrepancy suggests that other tissue (i.e., interstitium and/or lymphatics) intervenes in between the SAS and the blood. The authors attributed this outflow of CSF to bulk flow as different sized macromolecular tracers (albumin and 150 kDa dextran) were recovered in equal measure [153]. An additional study by this group used an implanted cannula in a cervical lymphatic vessel of unanesthetized sheep and attributed around 32% of the outflow of radiolabeled albumin to lymphatics [217].

The Johnston group used a similar approach in unanesthetized sheep and came to a value of closer to 50% of the tracer recovered in lymphatic vessels cannulated in the cervical region and the thoracic duct [157]. This group then further improved this technique using mathematical models based upon mass balance calculations of the dynamics of ^125^I-HSA injected into the ventricles as compared to ^131^I-HSA injected intravenously to account for the loss of the tracers over time from the plasma. The data from these models indicated that 40–48% of the CSF outflow was occurring through lymphatic vessels, with the remainder assumed (but not shown) to occur through arachnoid villi [218]. Using a ligation approach in rats, a similar value was obtained [219]. These authors also tested the effect of increasing ICP in anesthetized sheep [220, 221]. Cervical lymph flow increased substantially at increasing ICP, but never reached the values seen in unanesthetized animals under normal pressure conditions. It is important to note that an unknown proportion of the cervical lymph flow will originate from non-CNS sources (e.g., nasal tissue, middle ear) which likely also exhibit increased lymph formation while the animals are conscious. The authors proposed that the drop of CSF outflow resistance that has been shown to occur with increasing ICP [54, 222] might be a function of an enhanced lymphatic transport of CSF.

Several groups have shown that experimentally obstructing the outflow at the cribriform plate leads to significant reductions in CSF clearance to the lymphatic system in the neck [220, 97, 108, 223]. Bradbury and Westrop, after sealing the plate with cyanoacrylate glue, found that the recovery of ^125^I-albumin in deep cervical lymph on one side of the rabbit was reduced from 14.8% to 1.9% [108]. One must note that this 90% reduction in tracer recovery to deep cervical lymph does not mean that 90% of total CSF lymphatic outflow is through the cribriform plate, as alternate routes were not assessed. In fact, further evidence from this group indicated that flow down the spine may act as an alternate efflux site when flow from the nasal region is reduced, as previously demonstrated by Galkin in dogs [97]. The group of Johnston inhibited transport through the nasal pathway by sealing the extracranial surface of the cribriform plate of sheep with glue or bone wax [220, 119, 223]. Importantly, this procedure led to significantly higher spikes in ICP and increased CSF outflow resistance in response to fluid infusions into the ventricles and, remarkably, even increased ICP during homeostatic conditions when a ligation of the SAS of the spine was performed to block flow into this compartment. This indicates that, at least in sheep, the nasal route appears to be the most important for outflow from the cranial CSF space and that other potential outflow routes (through arachnoid villi, along other cranial nerves or to dural lymphatics) are not able to easily compensate.

## In vivo imaging

**Fig. 4:**
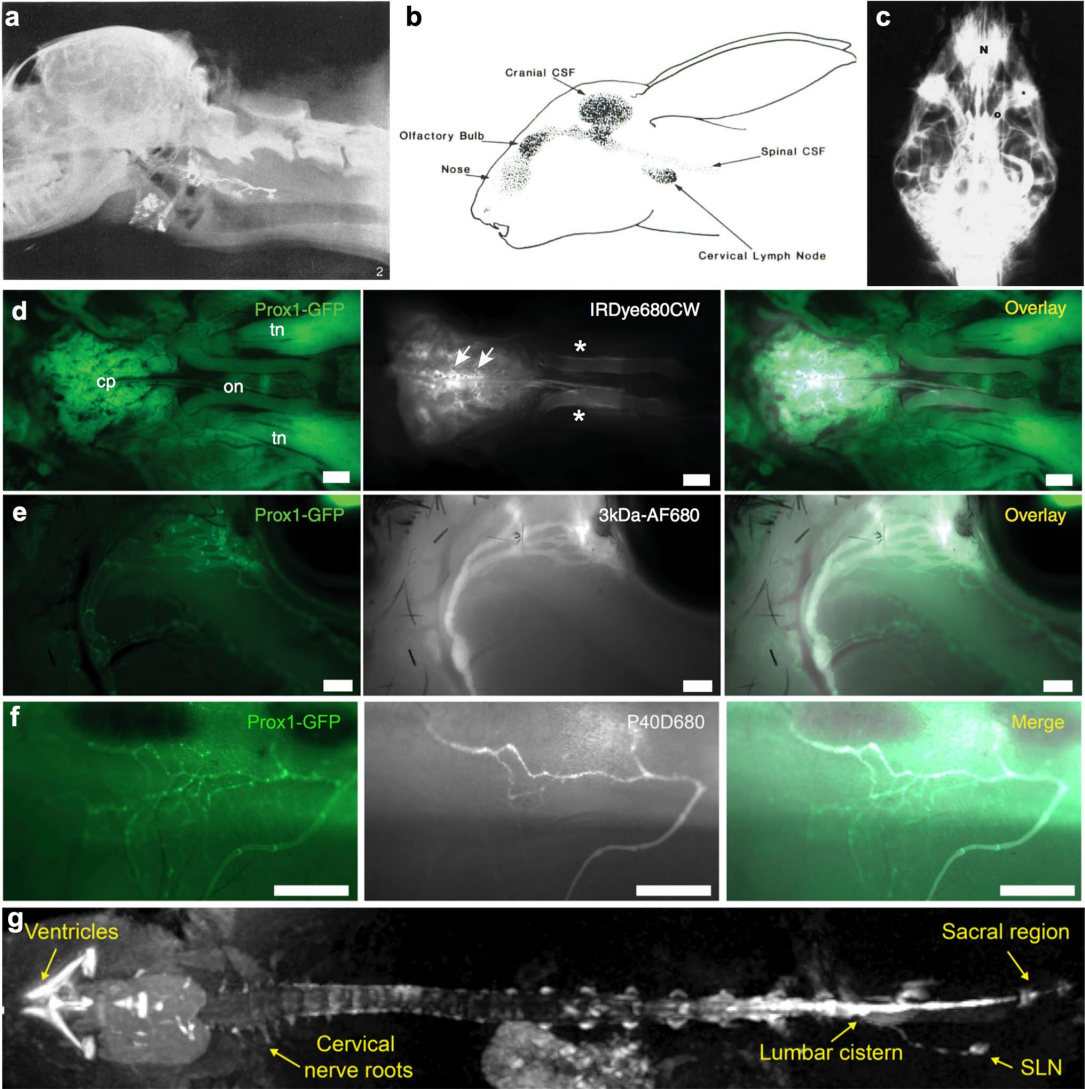
In vivo imaging techniques for assessment of outflow of CSF tracers.** a** Reproduction from Mortensen and Sullivan, 1933 [90]. The image shows a radiograph of a dog 6 h after injection into the cisterna magna with thorotrast. The contrast agent is seen within the lymphatic vessels and the superficial and deep lymph nodes. **b** An image reprinted from Pile-Spellman et al. [110] showing a scintigraphic image of a rabbit that was injected into the lateral ventricle with ^99m^Tc antimony sulfide. The sagittal image was acquired 4 h after injection and shows clear enrichment of radiotracer within the nasal region as well as in the deep cervical lymph node. **c** Inferior posterior radiograph of a cat skull 30 min after infusion of Isovist 300 contrast medium into the cisterna magna. Besides an accumulation in the nasal region (N), contrast medium (asterisk) is apparent in the orbital tissue and around the length of the optic nerve (O). Reproduced from [122] **d–f** Fluorescence microscopy imaging of the outflow of near-infrared fluorescent tracers from the CNS in Prox1-GFP transgenic reporter mice after intraventricular injection. Panel d indicates accumulation of IRDye680CW dye at the cribriform plate (cp, white arrows) and around the optic nerves (on, white asterisks). *tn* trigeminal nerves. In e, 3 kDa dextran labeled with AlexaFluor680 is shown exiting the orbit of the eye within Prox1-GFP^+^ lymphatic vessels. From this location the tracer drains into the mandibular (aka superficial cervical) lymph nodes (not shown). Panel f demonstrates an outflow of a 40 kDa pegylated near-infrared dye (P40D680) from the sacral region of the spine into Prox1-GFP^+^ lymphatic vessels. **g** Imaging with T1-weighted MRI of Gadospin D contrast agent showing spread to and outflow from the spine after low-rate infusion (0.1 μl/min) into the lateral ventricle. The outflow from the caudal end of the spine to the sacral lymph nodes is apparent. Panels d and e are reproduced from [130], while f and g are taken from [184]

The first in vivo imaging studies demonstrating CSF outflow were performed in the 1930s using X-ray [90, 98, 224]. Wustmann used a new contrast agent called thorotrast, which was a preparation of thorium dioxide suspended in a protective colloid, as a contrast agent for visualization of the SAS in living dogs. After injection, thorotrast found passage out of the cranium through perineural tissue spaces of the olfactory nerve towards the nasal mucosa. From this location the contrast agent was found in the lymphatic vessels at the angle of the mandible and in the deep cervical lymph nodes. In addition, there was contrast agent evident in the optic nerve sheaths as far as the papilla. From here, the thorium found passage into lymphatic collecting vessels in close proximity to facial veins to the submandibular lymph nodes. To a lesser extent, it was drained by the lymphatics accompanying the large blood vessels and cranial nerves at the base of the skull [98]. Mortensen and Sullivan found similar results in the same species, with thorotrast passing rapidly (within 30 min) to deep cervical nodes through a route that the authors determined was occurring along olfactory nerves towards the nasal mucosa and nasopharynx (Fig. 4a). Submaxillary lymph nodes also filled through a superficial network of lymphatic vessels that the authors were able to trace back to the bucco-nasal junction [90].

The technique was rapidly translated to the clinic [224, 225]. One published report in Germany by Jacobi and Lohr described clear delineation of the lymphatics of the mandible and pharynx and cervical lymph nodes in humans after cisternal administration of thorotrast [226]. The authors described an efflux along the olfactory and optic nerve sheaths. Unfortunately, no images are included in their report. Clinical utility was short-lived, however, as it was soon concluded from studies in cats, dogs and monkeys that intracranial administration of this contrast agent was harmful [227]. Although evidence of removal from the CSF spaces along cranial nerves was found, the overall clearance of thorotrast was slight. The development of hydrocephalus was a common result with thorotrast deposition in the basal cisterns and accumulation in macrophages of the SAS. However, radiographic contrast agents found continued application in animal studies. Schurr found efflux of pantopaque through the olfactory and optic nerve routes to submandibular and cervical lymph nodes in dogs [102]. Obliteration of the SAS over the cerebral hemispheres was induced by covering the brain with iodinated gauze which led to a fibrotic arachnoiditis. Despite the blockage of the suspected outflow sites at the arachnoid villi, there was no sign of hydrocephalus in the dogs until the optic and olfactory routes were also obstructed by the pantopaque contrast agent.

Di Chiro performed cisternography with radioionated serum albumin or autologous CSF in human patients, most of whom were suffering from epilepsy but would not be expected to exhibit any major deficiencies in CSF circulation [228, 229]. Although within one hour after lumbar injections, the radioactivity was clearly present in the basal cisterns, it took 12 h for the activity to be present over the convexity. At 24 h, high signal was present along the superior sagittal sinus. The author interpreted this finding as evidence for CSF streaming towards a site of outflow known to be rich in arachnoid granulations. However, the slow bulk movement of the tracer to this location would not agree with the expected rates of CSF turnover [230]. Other authors have interpreted these accumulations of radiolabeled proteins after cisternography to be occurring at the “dead ends of the CSF circulation”, rather than the major sites of absorption [231]. A more rapid clearance as observed in the basal cisterns would be evidence of faster turnover of CSF in these regions, which these particular authors speculated to be occurring through cerebral capillaries. In rabbits and cats, cisterna magna-injected 99Tc antimony sulfide colloid passed rapidly into the basal cisterns and was found within 5 min in the area of the olfactory bulb-cribriform plate (Fig. 4b) [110]. Cervical lymph node activity was first seen by scintigraphy after 30 min. Using a gamma counter to examine different tissues at the end of the experiment, the authors determined that a 1:2 ratio existed between the dose found in the deep cervical lymph nodes versus the liver. From this analysis, the authors estimated that 1/3 of the dose had exited along nerve routes to the lymphatic system, however, this calculation does not take into account outflow to other lymph node groups nor adjust for continued lymphatic transport of the radiocolloid through the lymph nodes to the blood.

Advancements in radiographic techniques and contrast agents have been utilized to further demonstrate lymphatic outflow in animals. Brinker et al., using dynamic X-ray microscopy in rats, found contrast media in the basal cisterns and nasal cavities soon after the start of infusion into the cisterna magna (Fig. 4c) [118]. Some minutes later, the contrast media highlighted the SAS of the optic nerve, the perilymphatic space of the inner ear, and the SAS above the cortical hemispheres and near the transverse sinuses. In another study using serial computed tomography scans, diatrizoic acid ester nanoparticles (240 nm diameter) were localized at the deep cervical lymph node of rabbits within 1 h of intraventricular injection. The authors estimated that 17% of the dose was present in the deep cervical lymph nodes at the peak enhancement time of 12 h after injection [232]. Similarly, Murtha et al. utilized contrast-enhanced CT scans to assess the CSF efflux routes in spontaneously hypertensive rats. The authors performed 3D scans every 5 min until 60 min after infusion of Ultravist contrast agent into the lateral ventricles and detected signals in the nasal tissue and deep cervical lymph nodes. The authors also detected spread of the contrast agent into the spinal SAS, but at no point could they detect signal at the superior sagittal sinus [127].

Fluorescence imaging techniques in rodents have recently been employed to visualize tracer outflow to lymphatic vessels. Mathieu et al. utilized a whole animal hyperspectral fluorescence imaging system to detect quantum dot efflux to the superficial cervical lymph nodes of mice [233]. Recently, researchers have been taking advantage of transgenic lymphatic vessel fluorescent reporter mice [234, 232]. One example of these mice, the Prox1-GFP mouse, was used by our group to map the outflow pathways of a CSF-injected pegylated near-infrared dye, a 40 kDa macromolecular tracer, to lymphatic vessels (Fig. 4d–e). We found tracer outflow along several cranial nerves, including olfactory, optic and facial nerves, with filling of lymphatic vessels immediately outside the skull [130]. Using non-invasive dynamic imaging, we demonstrated that tracers reached the collecting lymphatic vessels leading to the mandibular (or superficial cervical) lymph nodes before they were detectable within the systemic blood. The delay in signal enhancement that was apparent in the peripheral blood circulation (as measured at the saphenous vein) was consistent with the cannulation studies in larger animals performed by earlier investigators [56, 109, 114, 213]. Low molecular weight compounds exhibited similar transport dynamics to peripheral blood but with lower overall efflux, likely due to diffusion of these intraventicularly injected compounds across the ependyma into the brain and retention at various locations in the CNS. The dynamic near-infrared fluorescence assay to quantitively measure the CSF injected tracers in the systemic blood allows a sensitive noninvasive readout of CSF turnover through all potential efflux sites. We have used this approach to demonstrate a slower clearance of CSF in aged mice [130], a more rapid CSF turnover in awake as compared to anesthetized conditions [184] and a reduced efflux in glioblastoma-bearing mice [135].

Magnetic resonance imaging techniques hold great promise to evaluate the pathways of CSF flow in a dynamic, three-dimensional fashion with simultaneous anatomical visualization. Several investigators have explored the outflow of CSF using MRI techniques in animal studies. In an early study, Di Chiro et al. injected Gadolinium-DTPA into the cisterna magna of normal dogs and were able to visualize the efflux of the contrast agent across the cribriform plate into the nasal submucosa [236], replicating an earlier study in the same species using cisternograms [105]. The authors characterized this finding as CSF rhinorrhea, a condition in which CSF fluid leaks into the nasal cavities, however, they likely were visualizing the normal CSF efflux in this species. Muldoon et al. used Combidex, a dextran coated ultrasmall superparamagnetic iron oxide (USPIO) contrast agent injected into the lateral ventricles of rats [237]. They detected by MRI an accumulation, confirmed by histological methods, in both superficial and deep cervical lymph nodes by 2 h after injection. The authors, however, were unable to map the routes from the SAS to the lymph nodes. Another early MRI study injected the low molecular weight contrast agent, gadoteridol, into the cisterna magna of guinea pigs [121] and performed MRI scans every 30 min under different ICP conditions. The authors visualized and quantified a clear enhancement of the nasal (sub)mucosa and periorbital regions that was observed at earlier time points and seen at increased intensity when ICP was elevated. Almost no enhancement was observed in proximity to the superior sagittal sinus throughout the 4 h experiment.

More recent studies have utilized T1-weighted MRI and gadolinium-labeled contrast agents to map CSF flow pathways and efflux routes in rodents. Although not focused on determining the CSF efflux sites, a study by Gakuba et al. in isoflurane-anesthetized mice clearly shows enhancement of Gd-DOTA in what appears to be a continuous pathway through the cribriform plate, to the nasal (sub)mucosa and nasopharyngeal region to the deep cervical lymph nodes within 5 min of cisterna magna injection [238]. A study from our laboratory using gadolinium-labeled macromolecules injected into the cisterna magna has also demonstrated this rapid efflux route through the cribriform plate, as well as enhancement of contrast agent around the optic nerves and jugular foramina [135]. In a recent publication, however, Ahn et al. have concluded from MRI in rats that efflux routes at the base of the brain, presumably through meningeal lymphatic vessels, are the major drainage route to the deep cervical lymph nodes [160]. As mentioned before, this study did not evaluate the perineural routes through the nasal and optical regions. In fact, the positioning of the MRI coil did not appear to allow an assessment of these efflux routes. Therefore, the relative proportion of cranial CSF efflux through routes at the base of the skull versus perineural routes still needs to be determined.

It is to be expected that tracer injections, particularly when performed in a bolus fashion at high volumes and/or within short time periods, could alter the physiological flow patterns of CSF. This appears to be particularly apparent when injections are made into the intrathecal space of the spine [129, 131, 239] where a volume-dependent cranial spread of imaging tracers over time can be visualized. We have attempted to limit these effects by infusing MRI contrast agents in the lateral ventricle of mice using an indwelling cannula at rates (0.1 μL/min) far lower than estimated rates of CSF production in mice (~ 0.35 μL/min) [5]. Using this protocol, near-infrared imaging results were validated that demonstrate a caudal-directed flow and efflux of CSF at the sacral end of the spine to the draining sacral and deep iliac lymph nodes (Fig. 4f-g) [184]. Although MRI contrast agent showed accumulation at nerve roots along the length of the spine, there was no obvious efflux to lymphatics at any other level of the spinal cord. Further studies are needed to assess the anatomical differences at the nerve roots of the sacral region compared with other regions of the spine.

## Evidence from humans

Unlike in animals, evidence of defined CSF outflow pathways in humans is lacking. Early studies involving tracer injections in cadavers have provided evidence for routes through arachnoid granulations [2] and to the nasal submucosal lymphatics [2, 93, 94]. More recently, Lowhagen et al. filled India ink into the olfactory groove of cadavers without neurological disease and traced it through the cribriform plate along perineural routes [117], while Johnston et al. injected Microfil into the SAS of cadavers and could demonstrate filling of lymphatic vessels within the nasal submucosa [124].

Other insights on CSF outflow in humans have come from neuropathological assessments. Since arachnoid villi have long been considered the major efflux site, the morphology of these structures has been examined post mortem in cadavers of patients that have died from clinical conditions, such as intracranial hemorrhage or meningitis. In one of the first studies of this nature, post mortem evaluation of arachnoid granulations demonstrated infiltration of inflammatory cells in meningitis and red blood cells after hemorrhage [240]. The authors speculated that these pathological manifestations would impair the drainage function of these structures. Several authors have since confirmed that erythrocytes will accumulate in arachnoid projections after subarachnoid hemorrhage [241–243]. On the other hand, Lowhagen found red blood cells and iron-laden macrophages in abundance in the perineural spaces of the olfactory nerves and in the nasal submucosa after subarachnoid hemorrhage [117]. Another study identified red blood cells in deep cervical lymph nodes in a patient who died a few days after subarachnoid hemorrhage [244]. Red blood cells were also found to fill the SAS of the optic nerve. Although intact erythrocytes were not found within the orbital tissue outside of the nerve sheath, blood breakdown products, such as hemosiderin could be located at this location with a Berlin blue staining [244]. Caversaccio et al. found a significantly higher incidence of deep cervical lymph nodes containing iron in patients who had died from intracerebral hemorrhage versus cadavers without intracranial lesions [245].

In a provocative new study from a Spanish group, the authors utilized nasal swabs in an attempt to identify biomarkers in patients that had suffered from stroke. The authors were able to differentiate patients that had suffered from hemorrhagic stroke compared with ischemic stroke by the amount of iron in the nasal exudate. Although this study was preliminary, consisting of samples from only 20 patients, it serves as in vivo clinical evidence that CSF waste products may be cleared through nasal pathways, at least during pathological conditions [246].

Since the rediscovery of dural lymphatic vessels there has been a renewed push to image potential CSF outflow pathways in humans. These studies thus far have not been conclusive. Absinta et al. found that dural lymphatics could be visualized with MRI alongside the SSS in humans and primates [163]. These authors found that a low molecular weight contrast agent injected intravenously showed uptake into the lymphatics, while macromolecular contrast agents stayed confined to the blood vessels of the dura. Thus, while this study demonstrated that these vessels were functional, they were shown draining the dura mater interstitial fluid, rather than CSF. Another MRI study was able to visualize these vessels without contrast agent and found using a time-of-flight angiography technique that the lymphatic vessels demonstrated flow opposite to the flow of blood within the SSS. Thus, these vessels appeared to be draining towards the olfactory region [247].

De Leon et al. performed intravenous injection of two different low molecular weight radiotracers known to cross the CNS barriers and were able to highlight a potential efflux of CSF through the cribriform plate. Dynamic imaging with PET was superimposed on MRI images from each patient and showed clear enhancement of the superior nasal turbinates [248]. Eide et al. performed MRI imaging of deep cervical lymph nodes at several time points after intrathecal injection of gadobutrol in patients suffering from several types of CSF disorders. Lymph nodes showed a minor signal enhancement which peaked at 24 h [249]. A more recent study from this group has shown what appears to be an enrichment of signal at the parasagittal dura at 24 to 48 h after the intrathecal injection, which they interpret may be indicative of CSF flow towards lymphatic vessels in the dorsal dura [250]. These dynamics of signal enhancement near the superior sagittal sinus thus would be similar to those seen by Di Chiro using cisternography [228, 229]. It should be noted that low molecular weight gadobutrol also exhibits significant diffusion into the brain tissue and is thus not ideal for visualizing bulk flow CSF pathways [251]. Finally, a recent SPECT study by Verma et al. has provided evidence in healthy subjects for a delay in clearance dynamics after intrathecal administration of a ^99m^Tc-DTPA tracer [252]. The authors were able to visualize the spread of the tracer from the lumbar region of administration, to the basal aspect of the cranium and subsequently to the bladder for elimination. As the clearance from the blood of such a low molecular weight tracer would be very rapid, this delayed enhancement in the bladder indicates that there are no major efflux pathways from the SAS to blood vessels. Although the number of enrolled subjects was quite low, the exciting data points towards a prominent role for lymphatic outflow pathways for CSF egress in humans.

## Conclusions and open questions

**Fig. 5:**
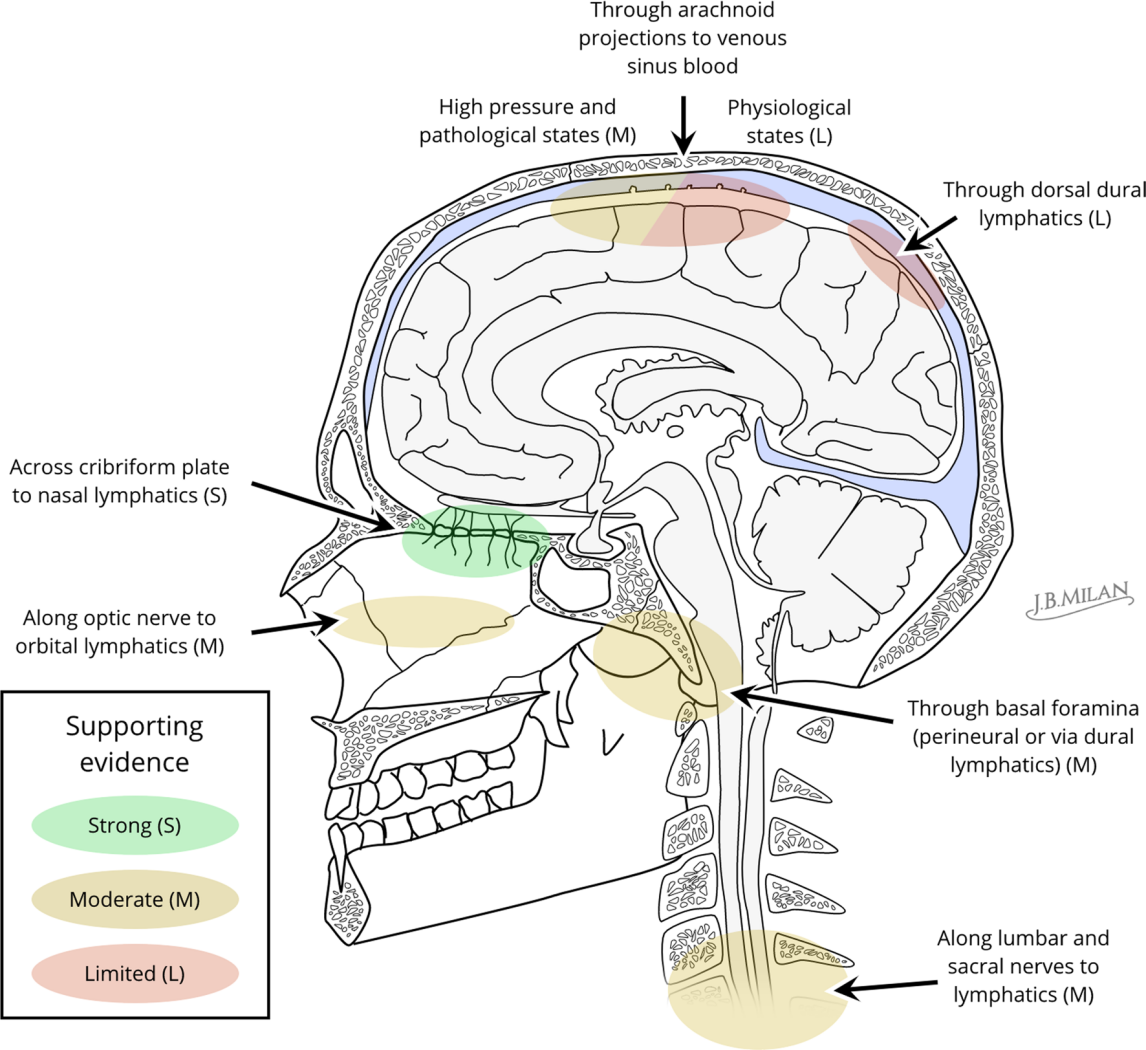
An overview of the evidence for routes of CSF outflow. A qualitative assessment of the level of supporting evidence for various CSF outflow pathways was made based on the number of published studies, uniformity of the results across different species and plausibility of the anatomical and physiological mechanisms. Experimental support appears to be strongest for an outflow of CSF at the cribriform plate with lymphatic vessels carrying the fluid and solutes to the deep cervical lymph nodes. Outflow also appears to be evident at the optic nerves and at the base of the skull through several possible foramina either in a perineural manner or through dural lymphatics. Evidence also exists for outflow from the spine to lymphatic vessels, particularly in the lumbar or sacral regions. CSF outflow has been detected in a limited number of studies in mice to the dural lymphatic vessels on the superior aspect of the skull. There is little support for an outflow to arachnoid villi or granulations under normal conditions, however, this route may become recruited during conditions of increased intracranial pressure. Scheme by Joachim Birch Milan

The existing evidence from animal and human studies for the various CSF outflow pathways is summarized in Fig. 5. This current review of the literature has highlighted the significant issues with the existing dogma that CSF efflux occurs through arachnoid projections to the venous system. Despite the lack of solid evidence for these structures draining CSF, the textbook concept has persisted almost in the same form as described by Lewis Weed in 1914. Even so, no consensus has been reached regarding a mechanism for outflow through arachnoid projections. There has been little anatomical study of these structures in recent decades with the exception of a few isolated studies, none of which directly support the notion that arachnoid projections are potential sites of significant fluid flow [253, 254, 250].

There has been intense focus on dural lymphatic vessels since their rediscovery in 2015 [144, 145, 160, 162, 179, 255–260]. Recent studies have found significant changes in CSF clearance, altered pathology in mouse models, or even cognitive defects, after ablation of the dural lymphatic vessels along the superior sagittal or transverse sinuses and it has been hypothesized that these effects are due to the dorsal lymphatic vessels playing a key role in CSF clearance and CNS immunosurveillance [179, 255, 259]. Although intriguing, it would appear that this theory is inconsistent with earlier experimental evidence and is further challenged by the findings in several studies that do not support the existence of a functional connection between CSF spaces and the interstitial space of the dura mater [3, 78, 130, 172]. This pathway, along with potential clearance mechanisms for brain interstitial fluid, are matters of controversy that need further exploration [16, 17, 261].

As is clear from this review, there is overwhelming historical evidence for perineural outflow pathways for CSF that have barely been acknowledged in several recent publications [160, 255, 258, 260, 262]. Of the three major proposed mechanisms for CSF outflow, the extracranial portions of cranial nerves and the region of the spinal nerves near the dorsal root ganglions are the only routes where barriers at either the arachnoid or endothelial cell layers have consistently been found to be lacking, allowing for bulk outflow as shown in physiological studies. Thus, the experimental and anatomical evidence in multiple species from rodents to primates point to perineural pathways being the most important pathway for CSF outflow. Ultimately, the question in humans will not be definitely answered until a method of sensitively and specifically imaging the egress of CSF can be established in the clinic. The alignment of evidence across mammalian species should, however, be sufficient to make clinicians and researchers critically question the textbook view of CSF outflow and to seek an improved understanding of the clearance pathways to the lymphatic system and the potential implications of these connections to the periphery for multiple neurological disorders.
